# Distribution of the schistosome intermediate snail host *Biomphalaria pfeifferi* in East Africa's river systems and the prevalence of *Schistosoma mansoni* infection

**DOI:** 10.1093/trstmh/trae115

**Published:** 2024-12-05

**Authors:** Victor O Magero, Sammy Kisara, Mbaruk A Suleman, Christopher M Wade

**Affiliations:** School of Life Sciences, University of Nottingham, Nottingham NG7 2RD, UK; Tropical and Infectious Diseases Department, Institute of Primate Research, P.O. Box 24481, Karen – 00502 Nairobi, Kenya; Tropical and Infectious Diseases Department, Institute of Primate Research, P.O. Box 24481, Karen – 00502 Nairobi, Kenya; Tropical and Infectious Diseases Department, Institute of Primate Research, P.O. Box 24481, Karen – 00502 Nairobi, Kenya; School of Life Sciences, University of Nottingham, Nottingham NG7 2RD, UK

**Keywords:** *Biomphalaria pfeifferi* snails, East Africa's river systems, human schistosomiasis, *Schistosoma mansoni*

## Abstract

**Background:**

There is a need for current and more detailed information on the distribution of *Biomphalaria pfeifferi* snails in East Africa's river systems. *B. pfeifferi* is arguably the most important snail intermediate host in the transmission of schistosomiasis, a disease ranked second to malaria in terms of tropical diseases of public health importance.

**Methods:**

We assessed the occurrence and geographical distribution of *B. pfeifferi* snails in Kenya, Uganda and Tanzania. Maximum entropy modelling was used to predict the potential distribution of *B. pfeifferi* snails and malacological surveys were conducted guided by MaxEnt predictions and information from previous studies. Malacological surveys were conducted at a total of 172 sites, including streams, rivers, dams, irrigation schemes and springs over a 3-y period from 2018 to 2020, with geospatial, ecological and physicochemical information recorded for each site.

**Results:**

*B. pfeifferi* snails were found at 23 of the 172 sites and inhabited a variety of habitat types. Of the 23 sites where *B. pfeifferi* snails were found, 15 (65.2%) were streams, 3 rivers (13.04%), 2 dams (8.7%), 2 springs (8.7%) and 1 an irrigation scheme (4.35%). *B. pfeifferi* abundance showed a significant positive correlation with increasing water temperature and decreasing water depth. In Kenya, *B. pfeifferi* snails were found around the Lake Victoria basin, the Mwea irrigation scheme and in parts of the former Eastern Province of Kenya. In Uganda, *B. pfeifferi* snails were found in Jinja District, Ntoroko District and Soroti District. In Tanzania, *B. pfeifferi* snails were found in the Iringa, Tabora and Kigoma Regions. We observed moderate to high prevalence of *Schistosoma mansoni* infection, with *S. mansoni*–infected snails found at 11 of 23 sites and with an average prevalence of 24.9% at infected sites. In Kenya, *S. mansoni*–infected snails were found in the Lake Victoria basin (22.5% prevalence at infected sites) and the former Eastern Province (13.5% prevalence at infected sites). In Uganda, infected snails were found in Ntoroko District (100% infected) and Soroti District (20% infected). In Tanzania, infected snails were found in the Kigoma Region, with a prevalence of 10% at the infected site.

**Conclusion:**

This information on the distribution of *B. pfeifferi* snails and *S. mansoni* infection in East Africa's river systems can aid in developing better prevention and control strategies for human schistosomiasis. Regular surveys of the river systems for snail intermediate hosts followed by molecular detection of schistosome infection could form a basis for the development of a prompt and cost-effective surveillance system for schistosomiasis in the region.

## Introduction

Schistosomiasis is a neglected tropical disease caused by flatworms of the genus *Schistosoma*. The disease is ranked second only to malaria in terms of tropical diseases of public health importance^1^ and it is estimated that approximately 1 billion people globally are at risk of becoming infected, with approximately 250 million people currently infected.^2^ Approximately 200 000 deaths are attributable to the disease annually.^1^ In East Africa, it is estimated that approximately 40 million people are infected with schistosomiasis, with approximately 140 million at risk of infection.^3^ The disease has debilitating effects on individuals because it renders them unproductive due to disability and malaise.^2^ Schistosomiasis is catastrophic to school-age children because it prevents them from attending school due to physical incapacity associated with the disease, thus jeopardising their chances of having a brighter future.^1^ There are two forms of human schistosomiasis, intestinal schistosomiasis caused by *Schistosoma mansoni, Schistosoma japonicum, Schistosoma intercalatum, Schistosoma mekongi* and *Schistosoma guineensis* and urogenital schistosomiasis caused by *Schistosoma haematobium*.^1,2^  *S. mansoni* and *S. haematobium* are responsible for the majority of cases of human schistosomiasis in Africa.^4,5^

Snail intermediate hosts are important because they support the transformation of *Schistosoma* parasites into infective stages.^6^ In East Africa, both *Biomphalaria* and *Bulinus* snails act as intermediate hosts for *Schistosoma* parasites, with *Biomphalaria* responsible for the transmission of *S. mansoni* and *Bulinus* responsible for the transmission of *S. haematobium*. There are several species of *Biomphalaria* implicated in *S. mansoni* transmission in East Africa, including *B. sudanica, B. choanomphala, B. smithi, B. stanleyi, B. angulosa* and *B. pfeifferi*,^7^ but *B. pfeifferi* is arguably the most significant snail intermediate host for the transmission of *S. mansoni* in Africa because of its widespread distribution and its relatively high susceptibility to schistosome infections.^8^  *Biomphalaria* snails are found in lakes, rivers, streams, dams, irrigation schemes and springs across East Africa. But while extensive studies have been undertaken on the African Great Lakes,^9–12^ information on the distribution of *Biomphalaria* in East Africa's river systems is less well understood.

River systems need to be studied because human communities that live close to the rivers use these rivers for activities such as sand harvesting, washing clothes, washing utensils, fetching water, bathing, fishing and watering livestock. These activities expose the human communities to the risk of being infected with *Schistosoma* parasites that infest these waters. It can be argued that the risk populations of river systems interact with rivers more than the risk populations of lakes interact with lakes based on the fact that rivers are relatively easy to access compared with lakes. Despite this risk, less attention is currently being given to studying river systems by schistosomiasis research groups in East Africa compared with the attention that continues to be given to the ecosystems of Lake Victoria and Lake Albert.^9,12–17^

Previous studies of *B. pfeifferi* snails in East Africa's river systems have found the snails in few locations. In Kenya, *B. pfeifferi* snails have been found at Muthamo seepage, Matingani seepage, Mbondoni Dam, Kangonde Dam, Mwea East, Mwea West, Onsando Dam, Grogan Canal, Kamayoga stream, Asawo stream, Kwahoma stream and Martin's drain.^18^ Malacological surveys conducted by Opisa et al.^19^ at informal settlements of Kisumu City identified *B. pfeifferi* in rivers and streams leading to Lake Victoria. *B. pfeifferi* snails were also found at the Mwea irrigation scheme, Asawo stream and Mukou stream^20^ and a study by Buddenborg et al.^21^ identified *B. pfeifferi* snails from Kasabong stream (western Kenya). *B. pfeifferi* snails have also been reported at Kibwezi,^22^ as well as Chebunyo Dam (Bomet County) and Churo pond (Kirinyaga County).^23^  *B. pfeifferi* snails were also collected from Asawo River and Kasabong stream, in western Kenya.^24^ In Uganda, *B. pfeifferi* snails have been found in Arua District^25^ and Buliisa District.^22^ In Tanzania, *B. pfeifferi* snails have been found in the southern part of the country, around Gombe National Park^26^ and in Kilombero District.^27^ The abundance of snails in any geographical location is important because the greater the abundance of the snail intermediate host in any location, the higher the probability that human beings will come in contact with *Schistosoma* parasites and be infected.^26^

Intestinal schistosomiasis (caused by *S. mansoni*) has been reported to be endemic in several localities in East Africa.^3^ However, there is little information on the role that *Biomphalaria* snails play in the transmission of intestinal schistosomiasis outside of Lake Victoria and Lake Albert. An increase in schistosome infections in snail intermediate hosts leads to contamination of water bodies with active cercariae and human populations become more at risk of infection. Some of the localities and regions where intestinal schistosomiasis has been reported to be endemic, with few to no reports of snail intermediate hosts, include Lango Region, West Nile Region and Soroti District in Uganda^28,29^ and Kilimanjaro Region, Tanzania.^30^ There is therefore a need to extensively establish the distribution of *Biomphalaria* snails in East Africa's river systems and their significance as intermediate hosts in the transmission of schistosomiasis. A better understanding of their distribution and role in schistosomiasis transmission will influence decision-making in the development of effective and integrated strategies to combat transmission of human intestinal schistosomiasis.

*B. pfeifferi* is arguably the most significant intermediate snail host for the transmission of intestinal schistosomiasis in Africa,^8,10,31^ and our study therefore focuses on this important host. We assess the occurrence of *B. pfeifferi* snails and their current geographical distribution in East Africa's river systems and determine the prevalence of *S. mansoni* infection in these snail populations. We also assess habitat, physicochemical and ecological parameters associated with the snails.

## Methods

### Ecological modelling and evaluation

Ecological modelling was done using MaxEnt,^32^ a machine learning algorithm that is used to predict the potential distribution of species based on the principle of maximum entropy in a situation where there is incomplete information about the distribution of a given species and the environmental factors that promote its habitation. To conduct maximum entropy species distribution modelling, occurrence points data (sites where *B. pfeifferi* snails have been found previously) and environmental layers (bioclimatic factors and environmental factors that affect the distribution of species) are required. Occurrence records for *B. pfeifferi* snails were obtained from the Global Biodiversity Information Facility (GBIF) database (https://www.gbif.org/). A total of 100 occurrence records were retrieved from the GBIF (with the *Biomphalaria* species identified in these studies using a combination of morphological and molecular methods). Bioclimatic variables were obtained from WorldClim.org and soil type and land cover variables were obtained from respective databases (soil type variables were derived from https://www.isric.org/explore/soil-geographic-databases and land cover variables were derived from http://2016africalandcover20m.esrin.esa.int/download.php).

### Malacological surveys

MaxEnt predictions (Supplementary Figure S1) and past knowledge on the distribution of *B. pfeifferi* snails in East Africa's river systems guided where we conducted our malacological surveys. Additionally, we visited water bodies in regions that have previously been reported as having cases of intestinal schistosomiasis, on the basis that there must be snail hosts in those localities that transmit the disease.

**Figure 1.**
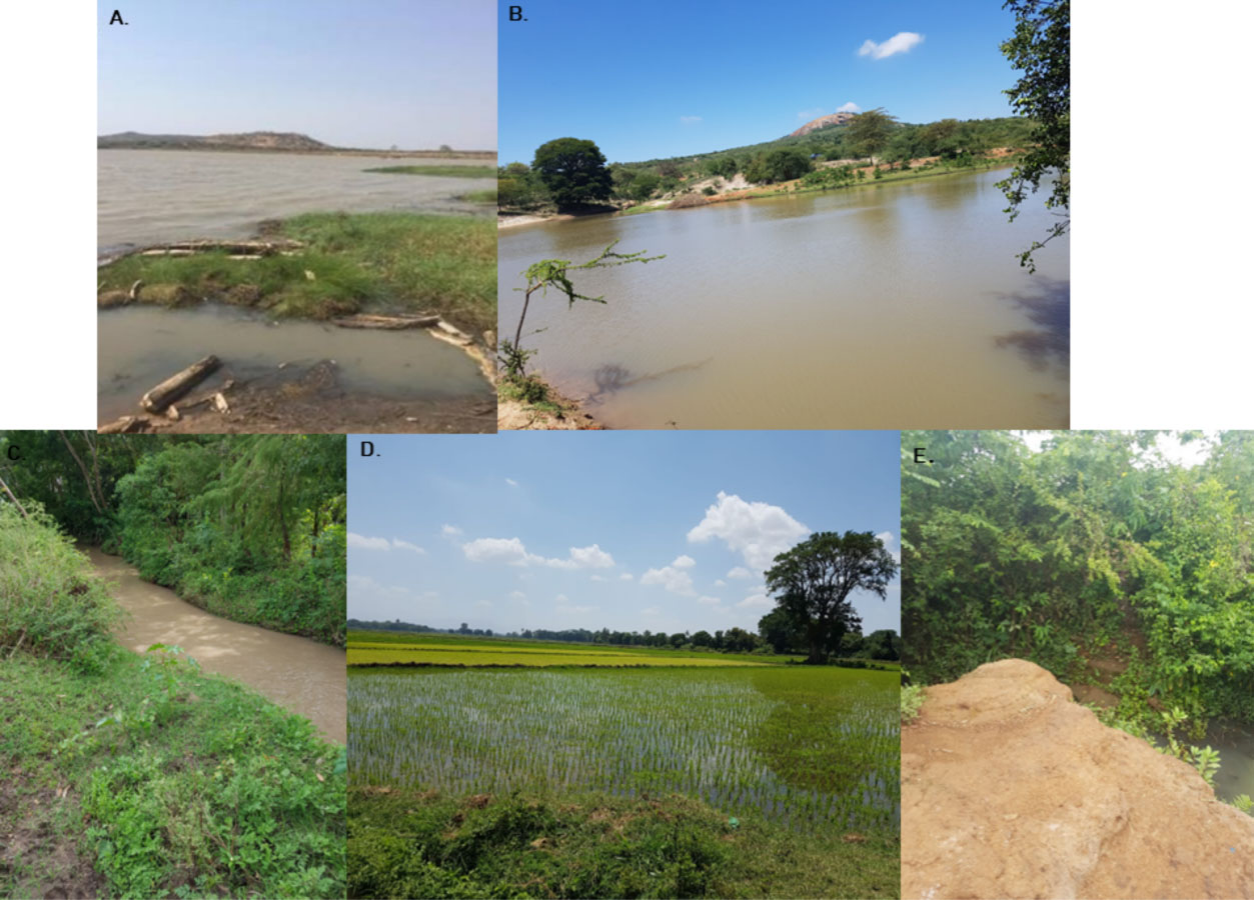
Different types of habitats from which the snails were sampled: (A) river (Ibulwa River, Tanzania), (B) dam (Kangonde Dam, Kenya), (C) stream (K’Otieno stream, Kenya), (D) irrigation scheme (Mabogini irrigation scheme, Tanzania), (E) spring (Opiyai Angorom, Uganda).

**Figure 2.**
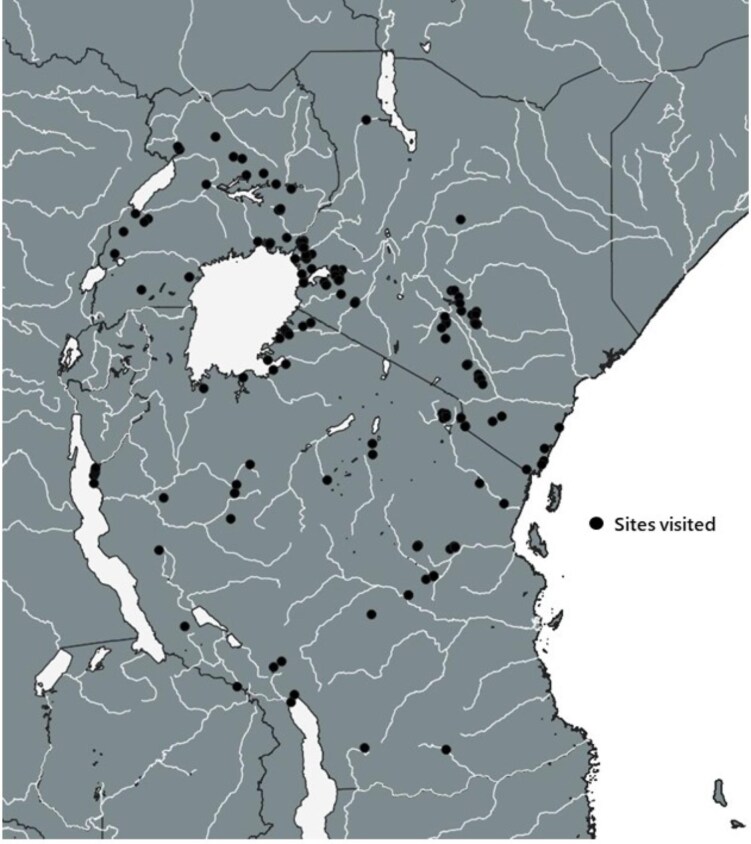
Map of East Africa showing sites visited for purposes of malacological surveys of *B. pfeifferi.*

Malacological surveys were conducted across Kenya, Uganda and Tanzania in search of *B. pfeifferi* snails. All collections were undertaken jointly by SK and VOM, with sampling undertaken from January to March and November to December 2018, January to March 2019 and January to March 2020. These months were selected as more suitable for sampling snails because historically they are months with light rains (not extreme weather conditions such as floods and drought). Various types of habitats were visited during the malacological surveys, including rivers, irrigation schemes, dams, streams and springs (Figure 1), with surveys undertaken at 72 sites in Kenya, 23 sites in Uganda and 77 sites in Tanzania (Supplementary Table S1 and Figure 2). Each site was visited once and only one malacological survey was conducted per site. The surveys involved searching water bodies for approximately 20 min, covering a distance of approximately 50 m and collecting all snails that were found, following procedures adopted by Standley et al.^33^ Collection of the snails was conducted using a handheld metallic scoop and geographic coordinates for each site were recorded using a handheld eTrex 10 GPS (Garmin, Kansas City, USA). We aimed to collect approximately 20–30 *B. pfeifferi* snails from each site where snails were found.

### Identification of *B. pfeifferi* snails

Snails were initially identified using established identification keys,^34–36^ with *B. pfeifferi* snails identified to the species level based on their morphological characters, including the general shape of the shell, shape of the whorls, number of coils and shape of the aperture. *B. pfeifferi* snails have a semi-oval, not circular shell; the shell has about 3–4 whorls, a diameter of 8–12 mm and a wide aperture. Snails were transferred to a collection jar then thoroughly washed and placed in falcon tubes while in the field. To preserve the snails, absolute ethanol was added to the tubes to a volume double that occupied by the snails. Guidelines for collecting molluscs and previous malacological studies suggest that storing molluscs in absolute ethanol is the best approach to preserve the snails following fieldwork.^9,23^

Molecular methods were then used to confirm the identity of the collected snails, with an average of 10–12 individuals from each snail population used in the molecular identification process. DNA extraction was undertaken using the cetyltrimethylammonium bromide (CTAB) method,^37^ using a small tissue slice from the head–foot region measuring approximately 2 mm × 2 mm. Polymerase chain reaction (PCR) was conducted using Bioline polymerase (BIO-21105) in combination with LCO (5′-GG TCAACAAATCATAAAGATATTGG-3′) and HCO (5′-TA AACTTCAGGGTGACCAAAAATCA-3′) primers developed by Folmer et al.^38^ which amplify a region of the cytochrome oxidase 1 (CO1) gene that is routinely used for snail identification. PCRs were carried out in a reaction buffer containing 2.5 mM MgCl_2_, 0.2 mM each dNTP, 0.2 µM of each primer, 1 unit of polymerase enzyme and 1 µl of template DNA from the snail extract. PCR cycling conditions involved an initial denaturation step of 2 min at 95°C, followed by 40 cycles at 94°C for 45 s, 55°C for 1 min and 73°C for 1 min and a final extension step at 73°C for 7 min. A negative control (no DNA) was included in all experiments. PCR products were run on a 1.5% agarose gel stained with ethidium bromide for verification of the amplifications and PCR products were sent to Macrogen Europe for purification and Sanger sequencing.

Sequence trace data were checked and edited in Finch TV^39^ and CO1 sequences aligned using Seaview version 5.0.^40^ We confirmed the identity of the snails collected by ‘BLASTing’ our sequences against GenBank and including them in a phylogenetic tree alongside representatives of African *Biomphalaria* snails available on GenBank. Phylogenetic trees were constructed in PhyML version 2.4.4 using the maximum likelihood method, using the GTR+G model and with bootstrap values based on 1000 replicates.^41^ The tree was rooted on *Biomphalaria glabrata* (accession number MK395976 CO1) following the phylogenetic tree of DeJong et al.^42^

### Geospatial, physicochemical and ecological parameters

Geospatial information, physicochemical parameters and ecological parameters associated with the sites were recorded. These parameters included vegetation type, water temperature and pH. Water temperature and pH were recorded using a portable water meter (Hanna Instruments, Møllevænget, Sweden). Water depth was recorded using a 1-m ruler and water velocity was recorded using a flowmeter (OTT HydroMet, Kempten, Germany). Additionally, we noted and recorded the soil type for each site. Supplementary Table S1 lists all the sites that were visited for malacological surveys, including their respective geographical coordinates, altitude, ecological information and physicochemical information.

Maps showing sites visited and sites where *B. pfeifferi* snails were found were created by QGIS version 3.12.3, with the name of the site, geographical coordinates, altitude, availability of snails, snail numbers and ecological and physicochemical parameters for each site recorded. To determine ecological and physicochemical factors associated with snail abundance, the Gaussian log function was used. In this function, *B. pfeifferi* snail abundance was used as an outcome while independent variables were the physicochemical and ecological parameters. Negative binomial generalised linear mixed models (GLMMs) were fitted in R version 4.1.0 (R Foundation for Statistical Computing, Vienna, Austria) using the package glmmTMB to test the association between snail abundance and parameters. Comparisons were made on all 172 sites, not just the 23 sites where *B. pfeifferi* snails were found.

### Molecular detection of *S. mansoni* infections in *B. pfeifferi* snails

The presence of *S. mansoni* infection in *B. pfeifferi* snails was determined using the PCR method developed by Lu et al.^24^ for the *ND5* gene (primers ND5F 5′-ATT AGA GGC AAT GCG TGC TC-3′ and ND5R 5′-ATT GAA CCA ACC CCA AAT CA-3′). Where numbers permitted, 10 snails were examined for *S. mansoni* infection at each site. PCRs were carried out in a reaction buffer containing 3.0 mM MgCl_2_, 0.2 mM each dNTP, 0.2 µM of each primer, 1 unit of polymerase enzyme and 1 µl of template DNA from the snail extract. The PCR cycling conditions were as follows: 30 cycles, each cycle consisting of 95°C for 1 min, 58°C for 1 min and 72°C for 30 s. A positive control containing pure *S. mansoni* DNA was used in each experiment. Two negative controls (Master mix with no DNA and Master mix with an *S. mansoni*–negative snail extract) were also included in the experiments. PCR products were run on a 1.5% agarose gel (stained with ethidium bromide) for 1 h at 100 V with gels visualised using an ultraviolet transilluminator to confirm amplified products.

## Results

### Presence and occurrence of *B. pfeifferi* snails

**Figure 3.**
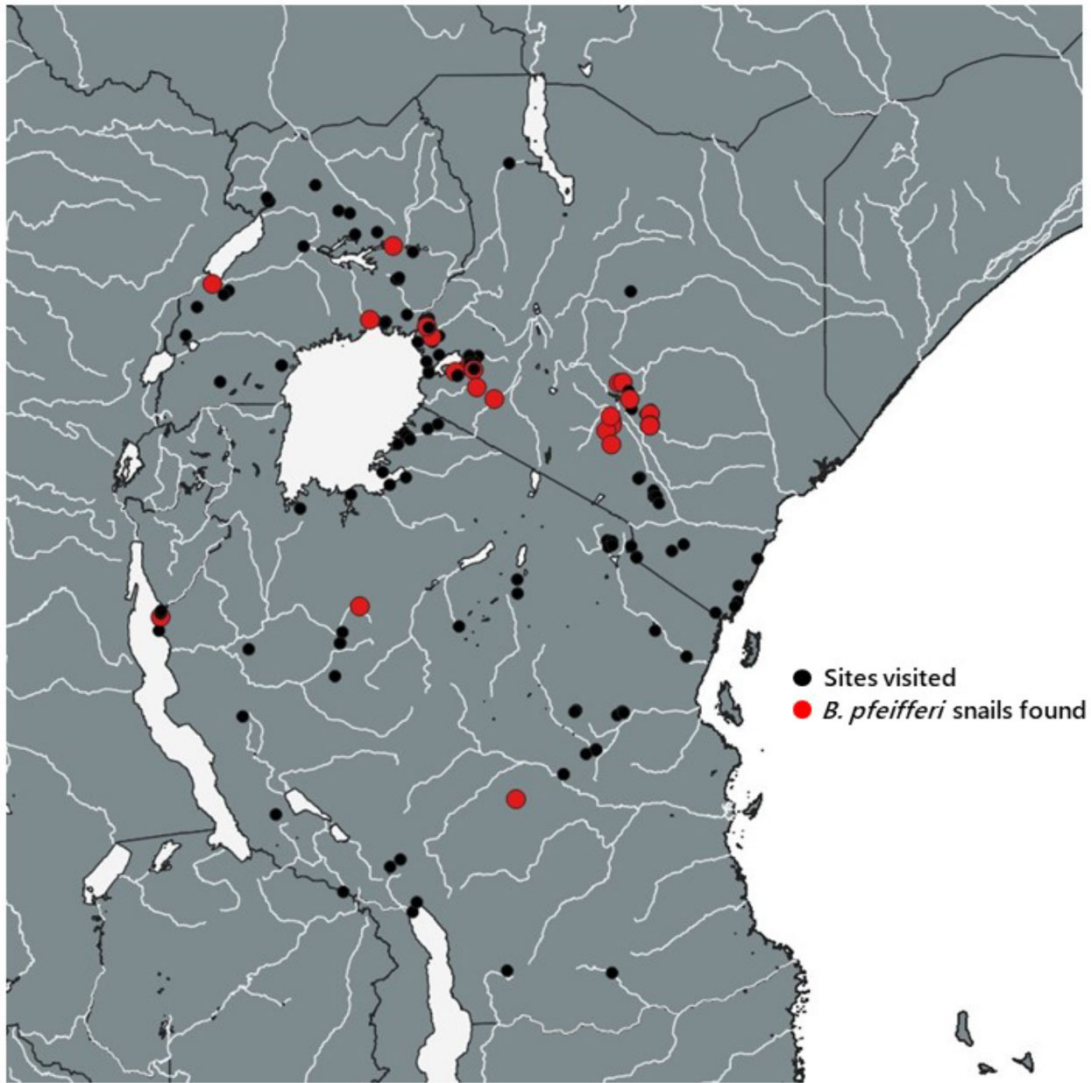
Map of East Africa showing sites where *B. pfeifferi* snails were found.

Incorporation of our *B. pfeifferi* CO1 sequences into a molecular phylogeny confirmed that all sequences fell within the *B. pfeifferi* clade (Supplementary Figure S2). *B. pfeifferi* snails were found at 23 of the 172 sites surveyed (Table 1, Supplementary Table S2 and Figure 3). The snails inhabit a variety of habitat types; 15 of the 23 sites where *B. pfeifferi* were found were streams, 3 of 23 were rivers, 2 of 23 were dams, 2 of 23 were springs and 1 of 23 was an irrigation scheme. Of the 23 sites, 17 were in Kenya, 3 in Uganda and 3 in Tanzania. Images of *B. pfeifferi* shells from the East African collection sites are shown in Supplementary Figure S3.

**Table 1.** Sites visited where *B. pfeifferi* snails were found in East Africa's river systems.

Site	Geographic coordinates	Altitude (m)	Type of habitat	Water temperature (°C)	pH	Water depth (cm)	Water velocity (cm/s)	Soil type	Vegetation
Matingani (Kenya)	−1.164, 38.005	1184	Stream	23.2	5.2	23.7	4.3	Clay	Yes
Kiangangi (Kenya)	−0.593833, 37.341972	1365	Irrigation scheme	24.5	8.2	17.5	6.5	Silt	Yes
Kalundu (Kenya)	−1.35616667, 38.00611111	1216	Stream	20.4	7.3	23.1	13.5	Sandy	Yes
Kwase (Kenya)	−1.29883333, 37.35722222	1568	Stream	19.3	6.8	19.3	16.5	Silt	Yes
Mutanga (Kenya)	−1.35758333, 37.35472222	1484	Stream	25.7	8.2	25.1	18.3	Silt	Yes
Itua (Kenya)	−0.62888889, 37.54	653	River	25.4	8.3	28.3	38.2	Silt	Yes
Tulimiumbu (Kenya)	−0.91133333, 37.65833333	1007	Stream	21.3	6.8	23.1	16.5	Sandy	Yes
Musilili (Kenya)	−1.45023611, 37.2575	1330	Stream	24.6	8.3	25.2	18.5	Silt	Yes
Kakulutuine (Kenya)	−1.20472222, 37.33055556	1188	Stream	25.3	7.6	21.7	15.3	Silt	Yes
Mukou (Kenya)	−1.68488889, 37.34472222	1319	Stream	25.9	9.2	19.2	19.5	Silt	Yes
Ambururu (Kenya)	0.15163889, 34.27916667	1236	Stream	23.5	7.5	20.2	26.4	Silt	Yes
Rakite (Kenya)	0.32575, 34.19472222	1285	Stream	21.2	8.2	23.1	12.4	Silt	Yes
Asawo (Kenya)	−0.31817, 35.007	1232	River	24.5	6.8	28.6	23.7	Silt	Yes
Kodumo (Kenya)	−0.41068, 34.99455	1532	Stream	21.2	7.3	29.7	12.9	Silt	Yes
Kodongo (Kenya)	−0.444710, 34.681030	1548	Stream	16.2	6.9	26.7	15.4	Silt	Yes
Onsando (Kenya)	−0.71103, 35.0479	1926	Dam	25.4	6.7	42.6	4.2	Silt	Yes
Cheptuyet (Kenya)	−0.91062, 35.34843	1914	Stream	25.3	8.3	27.3	14.1	Silt	Yes
Walukuba (Uganda)	0.44258, 33.22391	1139	Stream	25.6	7.3	26.3	14.2	Silt	Yes
Opiyai (Uganda)	1.70238, 33.62261	1119	Spring	25.2	6.2	27.2	12.6	Silt	Yes
Ntoroko (Uganda)	1.05375, 30.53696	631	River	20.8	8.4	28.6	13.6	Silt	Yes
Utwigu (Tanzania)	−4.446061, 33.05096	1227	Stream	20.6	6.4	16.3	12.4	Silt	Yes
Bwawani (Tanzania)	−7.73513, 35.71792	1530	Dam	24.1	8.5	18.6	13.7	Silt	Yes
Mwamgongo (Tanzania)	−4.62742, 29.65162	676	Stream	25.9	6.8	28.1	13.7	Silt	Yes

*B. pfeifferi* snails were found at 15 of the 70 stream sites that were visited (21.4%), 3 of the 69 river sites visited (4.3%), 2 of the 18 dam sites visited (11.1%), 2 of 2 spring sites visited (100%) and 1 of 13 irrigation scheme sites visited (7.7%) (Table 1, Supplementary Table S2).

### Ecological and physicochemical parameters

*B. pfeifferi* snail abundance showed a statistically significant relationship with water depth, soil type and temperature (Table 2). Water depth was statistically significant in relation to *B. pfeifferi* abundance (p=0.000009366), with the relationship between water depth and snail abundance inversely proportional. The snails were found at sites that had a water depth of <30 cm, suggesting that *B. pfeifferi* prefer shallow water. Soil type was statistically significant to the abundance of *B. pfeifferi* snails (p=0.00328), with the relationship between soil type and snail abundance being directly proportional. Habitats with sandy soil had the fewest number of snails followed by habitats with clay soil, whereas habitats with silt soil had the greatest number of snails. Likewise, water temperature was statistically significant to the abundance of *B. pfeifferi* snails (p=0.01281), with the relationship between water temperature and snail abundance being directly proportional. High numbers of snails were found at sites with water temperatures that ranged from 21°C to 27°C. Low numbers of snails were found at temperatures >27°C and <21°C. The other variables (vegetation, pH and water velocity) did not show any statistically significant relationship with the abundance of *B. pfeifferi* snails (Table 2).

**Table 2.** Estimates of the effects of various ecological and physicochemical parameters on the abundance of *B. pfeifferi* snails in East Africa's river systems.

Variables	Estimate	Standard error	Confidence interval	p-Value
Intercept	−14.22040	6708.07881	−1.316181e+04 to 1.313337e+04	0.99831
Vegetation	20.71477	6708.07665	−1.312687e+04 to 1.316830e+04	0.99754
Temperature	0.26582	0.10680	5.650474e-02 to 4.751363e-01	0.01281*
pH	−1.13942	0.65625	−2.425645e+00 to 1.467961e-01	0.08252
Water depth	−0.26054	0.05878	−3.757510e-01 to −1.453202e-01	0.000009366***
Soil type	1.94599	0.66187	6.487477e-01 to 3.243228e+00	0.00328**
Water velocity	−0.02135	0.04343	−1.064718e-01 to 6.377874e-02	0.62308

Negative binomial regression GLMM test in the glmmTMB package in R version 4.2.2 was used for the 172 sites.

Statistical significance at *p<0.5, **p<0.05, ***p<0.005.

### Schistosome prevalence

**Figure 4.**
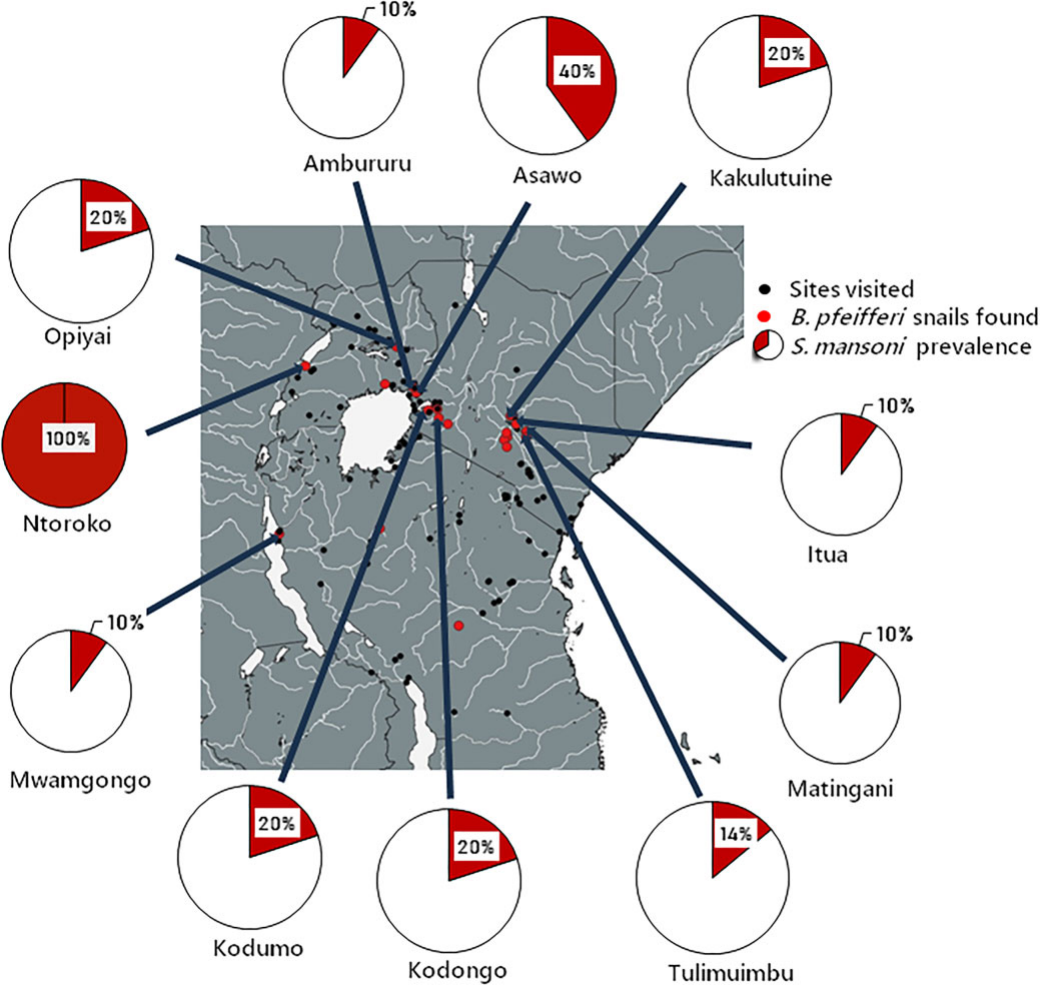
Map of East Africa showing the prevalence of *S. mansoni* in *B. pfeifferi* snails from East Africa's river systems. *S. mansoni* prevalence ranged from 10% to 100%.

*S. mansoni* was detected in 11 of the 23 *B. pfeifferi* populations (Table 3, Figure 4). In Kenya, we detected *S. mansoni* infection at Ambururu (1/10 snails [10%]), Asawo (4/10 [40%]), Itua (1/10 [10%]), Kakulutuine (2/10 [20%]), Kodongo (2/10 [20%]), Kodumo (2/10 [20%]), Matingani (1/10 [10%]), Mwamgongo (1/10 [10%]), Ntoroko (1/1 [100%]), Opiyai (2/10 [20%]) and Tulimuimbu (1/7 [14%]). In Uganda, *S. mansoni* infection was detected at Ntoroko (1/1 snails [100%]) and Opiyai (2/10 [20%]). In Tanzania, *S. mansoni* infection was detected at Mwamgongo (1/10 snails [10%]) (Figure 4). We observed low to moderate prevalence of *S. mansoni* infection, with an average prevalence of 24.9% at infected sites, ranging from 10% to 60%. In Kenya, *S. mansoni*–infected snails were found in the Lake Victoria basin and the former Eastern Province, with an average prevalence of 18% at infected sites. In Uganda, infected snails were found in Ntoroko District and Soroti District, with an average prevalence of 60% at infected sites. In Tanzania, infected snails were found in the Kigoma Region, with a prevalence of 10% at the infected site.

**Table 3.** Sites where *B. pfeifferi* snails were found and prevalence of *S. mansoni*.

Site	*S. mansoni* prevalence
Ambururu (Lake Victoria Basin, Kenya)	1/10
Asawo (Lake Victoria Basin, Kenya)	4/10
Cheptuyet (Lake Victoria Basin, Kenya)	0/10
Itua (Eastern Province, Kenya)	1/10
Ivuta (Eastern Province, Kenya)	0/10
Kakulutuine (Eastern Province, Kenya)	2/10
Kalundu (Eastern Province, Kenya)	0/10
Kiangangi (Mwea Irrigation Scheme, Kenya)	0/10
Kodongo (Lake Victoria Basin, Kenya)	2/10
Kodumo (Lake Victoria Basin, Kenya)	2/10
Kwase (Eastern Province, Kenya)	0/2
Matingani (Eastern Province, Kenya)	1/10
Mukou (Mwea Irrigation Scheme, Kenya)	0/10
Musilili (Eastern Province, Kenya)	0/10
Onsando (Lake Victoria Basin, Kenya)	0/10
Rakite (Lake Victoria Basin, Kenya)	0/5
Tulimuimbu (Eastern Province, Kenya)	1/7
Ntoroko (Ntoroko District, Uganda)	1/1
Opiyai (Soroti District, Uganda)	2/10
Walukuba (Jinja District, Uganda)	0/10
Bwawani (Iringa Region, Tanzania)	0/10
Mwamgongo (Kigoma Region, Tanzania)	1/10
Utwigu (Tabora Region, Tanzania)	0/10

## Discussion

### Geographical distribution of *B. pfeifferi* snails in East Africa's river systems

This study presents an extensive survey of *B. pfeifferi* snails in East Africa's river systems with sampling undertaken from 172 sites across Kenya, Uganda and Tanzania. *B. pfeifferi* snails are distributed across East Africa, outside of Lake Victoria and Lake Albert, with *B. pfeifferi* being found at 23 of the 172 sites sampled. *B. pfeifferi* inhabited a variety of habitat types. Of the 23 sites with snails, 15 (65.2%) were streams, 3 rivers (13.04%), 2 dams (8.7%), 2 springs (8.7%) and 1 (4.35%) an irrigation scheme. In Kenya, *B. pfeifferi* were found around the Lake Victoria basin (Kisumu County, Busia County, Siaya County, Kisii County, Homa Bay County and Nyamira County), at the Mwea irrigation scheme, Machakos County, Kitui County and Makueni County. In Uganda, the snails were found around the Lake Victoria basin and in the Albertine Region and Soroti District. In Tanzania, the snails were found in the Iringa, Tabora and Kigoma Regions.

Transmission of schistosomiasis is not possible in areas where there is an absence of snail intermediate hosts, therefore, understanding the distribution of snail hosts plays a role in prevention and control of the disease. Knowledge of the distribution of *B. pfeifferi* snails in East Africa's river systems can support surveillance responses in terms of detecting new transmission foci and potential schistosomiasis hotspots.

### The role of ecological and physicochemical parameters in the distribution of *B. pfeifferi* snails in East Africa's river systems

Ecological and physicochemical parameters have an influence on the distribution of *B. pfeifferi* snails. Water depth was established to be strongly significant in relation to *B. pfeifferi* snail abundance (p=0.000009366), with snail abundance decreasing with increasing water depth. A study by Magendantz^43^ found that water depth is an important factor as far as the distribution of snail intermediate hosts is concerned and that there is a correlation between the shallowness of a water body and the abundance of the intermediate hosts. In addition, Ofulla et al.^44^ showed a negative correlation between snail density and water depth in aquatic habitats of the Lake Victoria basin. Water is an important factor for the survival of snails, but too much of it decimates snail populations.^45^

Soil type was established to have a statistically significant association with the distribution of *B. pfeifferi* snails in East Africa's river systems (p=0.00328). Clayey soils have previously been noted to be important as far as survival of *Biomphalaria* snails. This is because they protect snails from desiccation during hot and dry seasons.^46^ It was established that sandy soils are not conducive for the habitation of *Biomphalaria* snails, as low snail abundance was found in water bodies rich in sandy soils. A study by Lydig et al.^46^ revealed that black cotton soils, rich in organic matter are favourable for the habitation of *Biomphalaria* snails. According to Ofulla et al.^44^ the substratum in any given habitat plays a role in the availability of snails and clay and silt soils constitute very important substrata favourable for the survival of *Biomphalaria* snails.

Temperature was established to be a crucial factor determining the geographical distribution of *B. pfeifferi* snails in East Africa's river systems (p=0.01281). Temperature as a climatic factor is very important for the survival of *Biomphalaria* snails.^47^ All sites with temperatures >30°C had no snails. Likewise, all sites with temperatures <15°C had no snails. Sites with a temperature range of 19.1°C–21.3°C had <20 snails, whereas sites with a temperature range of 23.2°C–26.1°C had >20 snails. Our findings showed that sites with temperatures >27°C had low snail densities compared with sites with temperatures of around 25°C. This suggests that temperature has a role to play in the abundance of *B. pfeifferi* snails in aquatic habitats. According to Yang et al.^47^ ideal temperature is important for the fecundity of snail intermediate hosts. Sturrock^48^ states that *Biomphalaria* snails are active at temperatures of 18°C–32°C, while reproduction and survival of the snails is optimum at a temperature range of 20°C–26°C. Optimum temperature is also important as far as the production of cercariae is concerned.^47^ Previous studies suggest that *Biomphalaria* snails are not as temperature tolerant as *Bulinus* snails and high temperatures discourage their habitation.^48^ This explains why *Bulinus* snails have previously been found in arid and semi-arid areas, whereas *Biomphalaria* snails are rarely found in such environmental conditions.^48^ Our findings agree with a study by Ofulla et al.^44^ that found *Biomphalaria* snails are less tolerant of temperatures >27°C. Habib et al.^10^ asserts that temperature is an important factor for *Biomphalaria* snails’ reproduction and survival and that temperature changes may alter the breeding, survival and geographical distribution of the snails. A positive correlation between water temperature and *B. sudanica* has also been reported.^14^ Temperatures higher than optimal values negatively interfere with egg production and the development of reproductive organs.^10^ It has been postulated that climate change, with subsequent global warming (with an increase in temperatures in freshwater bodies in the tropics and subtropics), may alter the geographical distribution of *Schistosoma* parasites and their snail intermediate hosts.^49^ Our study clearly demonstrates that temperature plays an important role in the geographical distribution of *B. pfeifferi* snails and *Schistosoma* parasites and supports the idea that climate change would be expected to influence the future occurrence of snail hosts and the incidence of disease.

The presence of vegetation in aquatic habitats has been reported to play a role in the distribution of freshwater snails.^50^ Although we did not find statistical significance between vegetation and *B. pfeifferi* abundance (p=0.99754), previous studies have reported that aquatic plants play an important role in the distribution and habitation of snails because they provide the necessary conditions for feeding, oviposition, breeding and shelter.^51^

Water velocity has also been established to have an influence on the distribution and abundance of freshwater snails,^52–54^ although we did not find statistical significance between water velocity and *B. pfeifferi* abundance in our study (p=0.62308). It has been reported previously that *Biomphalaria* snails are not found in sites with water velocities >30 cm/s.^55^ According to Appleton,^55^ water velocities can sweep *Biomphalaria* snails from one location to another, mostly during the rainy season. Floods are a bottleneck that has been confirmed to have a direct impact on the availability and distribution of *Biomphalaria* snails.^10^

Water pH has also been reported to have an influence on the distribution of *B. pfeifferi* snails,^45^ however, we did not find statistical significance between water pH and *B. pfeifferi* abundance in our study (p=0.08252). A study by Kazibwe et al.^14^ also suggests that pH has an influence on the distribution of African *Biomphalaria* snails.

### *S. mansoni* infection in *B. pfeifferi* snails in East Africa's river systems

Monitoring and surveillance of potential schistosomiasis transmission sites is important because this can help reduce morbidity and mortality related to the disease. Detection of *S. mansoni* infection in *B. pfeifferi* snails in East Africa's river systems is important because in doing so we can identify new transmission foci as well as persistent transmission hotspots. Traditionally, detection of *S. mansoni* infection in snail intermediate hosts has been determined by screening for cercarial shedding.^56^ However, PCR techniques have been proven to exhibit high levels of sensitivity and specificity as far as detection of parasitic infections is concerned.^56,57^

We used the *ND5*^24^ PCR technique to detect *S. mansoni* infections in *B. pfeifferi* populations. We observed *S. mansoni* infection at 11 of the 23 sites from which we collected *B. pfeifferi*, with an average prevalence of 24.9% (range 10–100%) at infected sites. We found relatively high levels of *S. mansoni* infection in areas around Asawo stream (western Kenya), Kodongo stream (western Kenya), Kodumo stream (western Kenya), Kakulutuine stream (eastern Kenya), Ntoroko (western Uganda) and Opiyai (northern Uganda). Snails collected from these locations had an *S. mansoni* prevalence of >20%.

In a study by Lu et al.^24^ on *B. pfeifferi* snails collected from the Asao River, Kenya (−0.318194, 35.006942), 47.6% of the snails were found infected by *S. mansoni*, courtesy of PCR amplification of the S*. mansoni ND5* gene. These results are very similar to our results, where we found 40% of *B. pfeifferi* snails at Asawo River, Kenya (−0.31817, 35.007) infected with *S. mansoni*. This is the same river, but at different locations 8 km apart; Lu et al.^24^ calls the river ‘Asao’, whereas we called it ‘Asawo’ based on what the locals told us and the name of the institution next to the river (Asawo Primary School).

A study by Bakuza et al.^26^ detected *S. mansoni* infections in 46.9–55.6% of *B. pfeifferi* snails collected from different sites within the Gombe National Park ecosystem, courtesy of the amplification of the internal transcribed spacer region of the ribosomal RNA gene. In our study, 10% of *B. pfeifferi* snails collected from Mwamgongo, which is in the Gombe National Park ecosystem, were found to be infected with *S. mansoni*.

The relatively high prevalence of *S. mansoni* in *B. pfeifferi* populations outside the Lake Victoria basin and Lake Albert region could be attributed to the fact that most schistosomiasis control efforts have focused on the Great Lakes Region^16,58–64^ and have neglected localities with potentially high levels of schistosome infections outside the Great Lakes Region. As a result, there is a likelihood that the number of *Schistosoma* parasites in the Great Lakes Region has decreased over the years while the numbers at other localities have remained the same or increased. The high level of compatibility between *S. mansoni* and *B. pfeifferi* when compared with other *Biomphalaria* species such as *B. choanomphala* and *B. sudanica* is another factor that could potentially explain high infection rates among the *B. pfeifferi* populations.^20^ Thus there is a need to shift attention of control efforts to potential endemic areas outside the Great Lakes Region.

Homozygosity (a loss of genetic diversity) exhibited by most *B. pfeifferi* populations is likely to be the major factor as to why *B. pfeifferi* snails are more susceptible to *S. mansoni* populations when compared with other African *Biomphalaria* snails. *B. pfeifferi* snails mostly inhabit environments that are prone to drought and floods and that often contributes to the snails experiencing recurrent size fluctuations and bottlenecks.^65^ Consequently, because of these recurrent size fluctuations, most *B. pfeifferi* engage in self-fertilisation as the preferred mode of reproduction (*B. pfeifferi* is capable of cross-fertilization). As a result, the genetic diversity in most *B. pfeifferi* populations is decreased. A study by Mutuku et al.^31^ investigated the compatibility of *S. mansoni* with *B. pfeifferi* in Kenya and found that persistent self-fertilisation and geographical isolation of *B. pfeifferi* populations could be major factors that contribute to greater compatibility of *S. mansoni* with *B. pfeifferi* populations. Furthermore, there are studies that assert that snail intermediate hosts that engage in outcrossing possess rare traits that confer a level of resistance to digenean infections, whereas for snail intermediate hosts that engage in self-fertilisation, the parasites are able to readily adapt and achieve high levels of infection.^66–68^

## Conclusions

This study showed that *B. pfeifferi* snails are widely distributed in East Africa's river systems and the distribution of the snails is influenced by ecological and physicochemical factors. Streams are the habitats preferred by the snails, followed by rivers, springs and irrigation schemes. Water depth, soil type and water temperature were established as the most important factors that influence the geographical distribution of the snails. *S. mansoni* infection was detected in 11 of the 23 sites where *B. pfeifferi* snails were found, with an average prevalence of 24.9% at infected sites. This study provides information on the distribution of *B. pfeifferi* snails in East Africa's river systems and contributes towards the body of knowledge on schistosomiasis and snail intermediate hosts. Although snail sampling was conducted extensively in East Africa, further sampling should be conducted because of the seasonality of the water bodies that are inhabited by the snails and the effects of floods and droughts on those habitats. We recommend follow-up in the geographical areas where *S. mansoni* infection was relatively high in the *Biomphalaria* snail populations by conducting diagnostic tests for the presence of *S. mansoni* infection in human subjects in areas and communities close to the habitats of the infected *Biomphalaria* snails. With this follow-up, we will be able to establish the prevalence of *S. mansoni* infection in human populations in the respective areas and communities and subsequently initiate relevant prevention and control strategies.

## Supplementary Material

trae115_Supplemental_File

## Data Availability

All data are provided in the manuscript. Sequence data are provided in GenBank (accession numbers PQ642945–PQ643167). [Note that sequences will be submitted to GenBank upon acceptance of the paper].
