# Supplementary material for: Distribution of the schistosome intermediate snail host Biomphalaria pfeifferi in East Africa's river systems and the prevalence of Schistosoma mansoni infection
Source: Trans R Soc Trop Med Hyg. 2024 Dec 5;119(3):253–65. doi: 10.1093/trstmh/trae115 (PMC11887620; doi:10.1093/trstmh/trae115)
Supplement: trae115_Supplemental_File [file trae115_supplemental_file.docx]

**SUPPLEMENTARY FILES**

**Supplementary Table 1:** GBIF Occurrence Records table.

| **COUNTRY** | **GBIF Catalogue Number/Occurrence ID** | **GEOGRAPHIC COORDINATES** |
| --- | --- | --- |
| Angola | NHMUK:ecatalogue:8168749 | (-9.300000, 16.100000) |
| Benin | ET_MBI_1559 | (10.200000, 2.100000) |
| Benin | ET_MBI_1563 | (6.400000, 2.200000) |
| Benin | 2019-10 | (9.300000, 2.600000) |
| Bostwana | 19000 | (-18.300000, 21.800000) |
| Bostwana | 10283 | (-18.400000, 21.900000) |
| Bostwana | 9035 | (-18.400000, 22.000000) |
| Bostwana | 52010 | (-18.900000, 22.400000) |
| Bostwana | 56691 | (-19.000000, 22.400000) |
| Bostwana | 59700 | (-19.100000, 23.400000) |
| Bostwana | 28139 | (-19.200000, 23.200000) |
| Bostwana | 64191 | (-19.500000, 24.100000) |
| Botswana | 52010 | (-18.900000, 22.400000) |
| Botswana | 21221 | (-19.500000, 23.100000) |
| Burkina Faso | MSB:Host:20665 | (11.800000, -0.500000) |
| Burundi | NHMUK:ecatalogue:8168777 | (-3.000000, 29.400000) |
| Burundi | NHMUK:ecatalogue:8168772 | (-4.200000, 29.600000) |
| Burundi | NHMUK:ecatalogue:8168778 | (-3.000000, 29.400000) |
| Cameroon | BE_RMCA_MOL.Gas.804806 | (4.500000, 9.300000) |
| Cameroon | NHMUK:ecatalogue:8173798 | (3.900000, 11.500000) |
| Chad | NHMUK:ecatalogue:9178255 | (19.100000, 20.500000) |
| Chad | NHMUK:ecatalogue:8168973 | (12.100000, 15.100000) |
| Congo | 6610884 | (-1.500000, 29.300000) |
| Congo | 132860 | (-7.000000, 23.900000) |
| Congo | BE_RMCA_MOL.Gas.804817 | (0.500000, 25.200000) |
| Congo | 186459 | (-7.000000, 23.900000) |
| Cote d’Ivore | CNFDOB020849 | (5.200000, -2.900000) |
| Egypt | MSB:Host:20664 | (29.700000, 31.200000) |
| Egypt | 558617-Mollusca | (25.700000, 32.600000) |
| Kenya | MSB:Host:25220 | (0.100000, 34.700000) |
| Kenya | MSB:Host:20705 | (-0.100000, 34.700000) |
| Kenya | MSB:Host:22342 | (0.200000, 34.400000) |
| Kenya | MSB:Host:22300 | (-0.300000, 35.000000) |
| Kenya | MNMK1064BP9 | (-0.400000, 34.200000) |
| Kenya | MSB:Host:24891 | (-0.300000, 35.200000) |
| Kenya | MNMK1064BP8 | (0.600000, 34.500000) |
| Kenya | MNMK1064BP10 | (0.700000, 34.500000) |
| Kenya | MNMK1064BP21 | (-1.200000, 36.900000) |
| Kenya | MNMK1064BP11 | (**-**1.300000, 37.300000) |
| Kenya | MNMK1064BP22 | (-1.400000, 36.700000) |
| Kenya | MNMK1064BP23 | (-2.700000, 37.300000) |
| Kenya | MNMK1064BP6 | (3.300000, 37.100000) |
| Kenya | MNMK1075BS3 | (-0.100000, 36.400000) |
| Kenya | MSB:Host:25074 | (0.200000, 34.300000) |
| Kenya | MSB:Host:24905 | (-0.300000, 35.000000) |
| Malawi | RMNH.MOL.511026 | (-13.500000, 34.000000) |
| Malawi | DMNS:Inv:38264 | (-15.800000, 35.000000) |
| Niger | 6619408 | (19.000000, 10.500000) |
| Nigeria | MSB:Host:20673 | (9.300000, 9.600000) |
| Senegal | GBML27974-19 | (14.200000, -14.600000) |
| Senegal | 172740 | (14.700000, -17.500000) |
| Senegal | NHMUK:ecatalogue:8535873 | (16.100000, -16.400000) |
| Senegal | NHMUK:ecatalogue:8171940 | (16.500000, -15.600000) |
| Senegal | GBML27974-19 | (14.200000, -14.600000) |
| Senegal | BE_RMCA_MOL.Gas.804931 | (16.400000, -15.900000) |
| Senegal | BE_RMCA_MOL.Gas.804949 | (16.500000, -15.900000) |
| Senegal | NHMUK:ecatalogue:8534742 | (15.900000, -15.900000) |
| Senegal | NHMUK:ecatalogue:8535404 | (16.100000, -16.400000) |
| Senegal | NHMUK:ecatalogue:8173479 | (16.300000, -15.800000) |
| Senegal | NHMUK:ecatalogue:8173565 | (16.400000, -15.700000) |
| South Africa | LIM 893Z | (-23.800000, 31.600000) |
| South Africa | GEN 155K | (**-**25.100000, 30.800000) |
| South Africa | ELMD06212 | (-32.200000, 29.000000) |
| Tanzania | MNMK1075BS15 | (**-**2.500000, 32.900000) |
| Tanzania | 84352 | (**-**3.100000, 37.400000) |
| Tanzania | GBML27890-19 | (-6.400000, 34.800000) |
| Tanzania | NHMUK:ecatalogue:8536591 | (-2.700000, 32.800000) |
| Tanzania | NHMUK:ecatalogue:8536623 | (-2.300000, 32.200000) |
| Tanzania | NHMUK:ecatalogue:8172704 | (-2.800000, 32.900000) |
| Tanzania | MNMK0408BC11 | (-2.100000, 32.900000) |
| Tanzania | 159078 | (-2.500000, 32.900000) |
| Tanzania | NHMUK:ecatalogue:8536489 | (-2.500000, 32.800000) |
| Tanzania | NHMUK:ecatalogue:8536295 | (-2.500000, 32.800000) |
| Tanzania | NHMUK:ecatalogue:8536370 | (-2.700000, 32.800000) |
| Tanzania | NHMUK:ecatalogue:8172605 | (-2.400000, 32.100000) |
| Uganda | RS_NaFIRRI_Macro Inverts_12346 | (0.100000, 32.600000) |
| Uganda | BE_RMCA_MOL.Gas.804913 | (-0.800000, 30.900000) |
| Uganda | GBML27983-19 | (1.300000, 32.300000) |
| Uganda | BE_RMCA_MOL.Gas.804543 | (-0.300000, 30.100000) |
| Uganda | 118593 | (-0.300000, 32.600000) |
| Uganda | 159084 | (0.800000, 30.100000) |
| Uganda | GBML27983-19 | (1.300000, 32.300000) |
| Uganda | 172097 | (1.800000, 31.700000) |
| Uganda | 124545 | (1.800000, 31.300000) |
| Uganda | 171168 | (0.000000, 30.100000) |
| Uganda | 171716 | (-0.400000, 29.600000) |
| Uganda | BE_RMCA_MOL.Gas.804500 | (0.400000, 30.200000) |
| Uganda | BE_RMCA_MOL.Gas.804552 | (0.400000, 30.300000) |
| Uganda | RS_NaFIRRI_Macro Inverts_443 | (0.400000, 33.200000) |
| Uganda | BE_RMCA_MOL.Gas.804567 | (0.400000, 33.300000) |
| Uganda | 137126 | (1.800000, 31.300000) |
| Uganda | BE_RMCA_MOL.Gas.804871 | (-0.500000, 31.200000) |
| Uganda | BE_RMCA_MOL.Gas.804479 | (0.700000, 30.300000) |
| Uganda | BE_RMCA_MOL.Gas.804870 | (-0.800000, 30.900000) |
| Uganda | RS_NaFIRRI_Macro Inverts_15370 | (1.800000, 31.300000) |
| Uganda | 171384 | (-1.200000, 29.700000) |
| Uganda | 171383 | (0.300000, 32.600000) |
| Uganda | 41128 | (0.400000, 29.900000) |
| Uganda | GBMLG9911-13 | (1.300000, 32.300000) |
| Zimbabwe | BE_RMCA_MOL.Gas.804643 | (-17.200000, 31.000000) |
| Zimbabwe | BE_RMCA_MOL.Gas.804634 | (**-**17.300000, 31.000000) |

**Supplementary Table 2:** Geographic coordinates, ecological data and physicochemical data.

| **Name of site** | **Coordinates** | **Altitude** | **Type of habitat** | **Water Temp (°C)** | **pH** | **Water depth (cm)** | **Water velocity (cm/s)** | **Soil Type** | **Vegetation**  **(Y/N)** | ***B. pfeifferi***  ***found* (Y/N)** |
| --- | --- | --- | --- | --- | --- | --- | --- | --- | --- | --- |
| Mbondoni (Kenya) | (-0.97325, 38.005) | 1121m | Dam | 31.2 | 7.3 | 92.5 | 2.9 | Sandy soil | N | N |
| Migwani (Kenya) | (-1.09305556, 38.02111) | 1116m | Dam | 29.1 | 6.2 | 75.4 | 3.1 | Sandy soil | N | N |
| Matingani (Kenya) | (-1.164, 38.005) | 1184m | Spring | 23.2 | 5.2 | 23.7 | 4.3 | Clay soil | Y | Y |
| Kangonde (Kenya) | (-1.07941667,  37.69166667) | 1183m | Dam | 31.2 | 8.3 | 86.4 | 3.7 | Sandy soil | N | N |
| Mutendea (Kenya) | (-1.33180556,  37.98166667) | 1098m | Stream | 29.1 | 7.3 | 35.2 | 11.5 | Sandy soil | Y | N |
| Kiangangi (Kenya) | (-0.593833,  37.341972) | 1365m | Irrigation scheme | 24.5 | 8.2 | 17.5 | 6.5 | Silt soil | Y | Y |
| Kitui (Kenya) | (-1.35616667,  38.00611111) | 1166m | Stream | 20.6 | 6.4 | 16.3 | 12.4 | Silt soil | Y | Y |
| Kalundu (Kenya) | (-1.35616667,  38.00611111) | 1216m | Stream | 20.4 | 7.3 | 23.1 | 13.5 | Sandy soil | Y | Y |
| Kalundu (Kenya) | (-1.36555556,  38.00277778) | 1214m | Stream | 20.1 | 8.2 | 19.4 | 12.1 | Sandy soil | Y | Y |
| Ikindu  (Kenya) | (-1.36733333,  38.03916667) | 1215m | Stream | 31.2 | 9.2 | 38.2 | 11.7 | Sandy soil | Y | N |
| Nzeu (Kenya) | (-1.37061111, 38.02416667) | 1138m | Stream | 29.1 | 7.2 | 42.1 | 12.2 | Sandy soil | N | N |
| Kwase (Kenya) | (-1.29883333,  37.35722222) | 1568m | Stream | 19.3 | 6.8 | 19.3 | 16.5 | Silt soil | Y | Y |
| Mutanga (Kenya) | (-1.35758333,  37.35472222) | 1484m | Stream | 25.7 | 8.2 | 25.1 | 18.3 | Silt soil | Y | Y |
| Chumbe (Kenya) | (-2.26511111, 37.81027778) | 950m | Stream | 32.1 | 7.9 | 19.3 | 17.6 | Sandy soil | N | N |
| Ikoyo (Kenya) | (-2.26613889, 37.81) | 994m | Dam | 31.2 | 8.4 | 42.1 | 5.7 | Sandy soil | N | N |
| Thange (Kenya) | (-2.47025,  38.06583333) | 776m | River | 14.1 | 6.8 | 59.6 | 34.2 | Sandy soil | Y | N |
| Thange (Kenya) | (-2.50158333,  38.02277778) | 777m | River | 15.6 | 7.3 | 72.4 | 35.5 | Sandy soil | Y | N |
| Thange (Kenya) | (-2.47536111,  38.09527778) | 775m | River | 15.2 | 8.3 | 71.3 | 32.6 | Sandy soil | Y | N |
| Kambu (Kenya) | (-2.536591, 38.118119) | 781m | River | 31.5 | 7.4 | 72.8 | 27.6 | Sandy soil | Y | N |
| Kambu (Kenya) | (-2.498523, 38.053190) | 782m | River | 30.9 | 9.3 | 69.2 | 29.4 | Sandy soil | Y | N |
| Loilopon (Kenya) | (-2.930847, 37.476999) | 1910m | Stream | 13.2 | 8.2 | 42.4 | 28.6 | Silt soil | N | N |
| Kambu (Kenya) | (-2.56630556,  38.11944444) | 779m | River | 20.9 | 9.3 | 43.6 | 29.3 | Sandy soil | Y | N |
| Itua (Kenya) | (-0.62888889,  37.54) | 653m | River | 25.4 | 8.3 | 28.3 | 38.2 | Silt soil | Y | Y |
| Thiba (Kenya) | (-0.77269444,  37.63888889) | 1009m | River | 15.3 | 7.2 | 65.2 | 37.4 | Sandy soil | Y | N |
| Tulimiumbu (Kenya) | (-0.91133333,  37.65833333) | 1007m | Stream | 21.3 | 6.8 | 23.1 | 16.5 | Sandy soil | Y | Y |
| Kitunene (Kenya) | (-0.92533333,  37.67583333) | 1221m | Dam | 15.2 | 9.2 | 96.3 | 4.1 | Sandy soil | N | N |
| Musilili (Kenya) | (-1.45023611,  37.2575) | 1330m | Stream | 24.6 | 8.3 | 25.2 | 18.5 | Silt soil | Y | Y |
| Kakulutuine (Kenya) | (-1.20472222,  37.33055556) | 1188m | Stream | 25.3 | 7.6 | 21.7 | 15.3 | Silt soil | Y | Y |
| Mukou (Kenya) | (-1.68488889,  37.34472222) | 1319m | Stream | 25.9 | 9.2 | 19.2 | 19.5 | Silt soil | Y | Y |
| Mtito (Kenya) | (-2.68713889, 38.16) | 464m | River | 21.2 | 8.1 | 38.2 | 25.2 | Sandy soil | Y | N |
| Kahoma (Kenya) | (-3.42088889, 37.68305556) | 845m | Stream | 15.4 | 8.2 | 37.2 | 17.6 | Silt soil | Y | N |
| Voi (Kenya) | (-3.39063889,  38.57861111) | 546m | River | 13.2 | 6.4 | 53.2 | 28.3 | Sandy soil | N | N |
| Jipe A (Kenya) | (-3.59861111,  37.77444444) | 705m | Stream | 31.5 | 7.3 | 63.4 | 34.6 | Sandy soil | Y | N |
| Jipe B (Kenya) | (-3.61477778,  37.77611111) | 706m | Stream | 32.1 | 8.2 | 58.9 | 38.4 | Sandy soil | Y | N |
| Jombo (Kenya) | (-3.50175, 38.37777778) | 903m | River | 14.2 | 7.1 | 75.2 | 41.2 | Sandy soil | N | N |
| Kasabong (Kenya) | (-0.15608333, 34.40583333) | 1182m | Stream | 29.3 | 8.3 | 52.1 | 17.2 | Silt soil | N | N |
| Abitha (Kenya) | (-0.16761111,  34.40583333) | 1184m | Stream | 29.7 | 9.1 | 15.2 | 19.7 | Silt soil | Y | N |
| Gera (Kenya) | (-0.46413889,  34.22916667) | 1152m | River | 31.2 | 8.2 | 48.2 | 34.6 | Silt soil | Y | N |
| Nyabanda (Kenya) | (-0.08961111,  34.28111111) | 1281m | Dam | 15.3 | 9.1 | 85.4 | 5.7 | Sandy soil | Y | N |
| Manyonge (Kenya) | (-0.06366667,  34.29166667) | 1289m | Stream | 14.2 | 7.8 | 36.2 | 32.7 | Silt soil | Y | N |
| Nyawita (Kenya) | (-0.43036111,  34.15472222) | 1149m | Stream | 13.5 | 9.2 | 39.2 | 13.6 | Silt soil | Y | N |
| Kosena (Kenya) | (0.09338889, 34.28) | 1281m | Dam | 15.3 | 8.1 | 75.1 | 5.6 | Sandy soil | N | N |
| Ambururu (Kenya) | (0.15163889,  34.27916667) | 1236m | Stream | 23.5 | 7.5 | 20.2 | 26.4 | Silt soil | Y | Y |
| Mbita (Kenya) | (-0.42180556,  34.20611111) | 1168m | River | 29.3 | 6.3 | 59.6 | 33.7 | Sandy soil | Y | N |
| Thogoye (Kenya) | (-0.06091667,  34.04305556) | 1134m | River | 31.2 | 7.2 | 60.2 | 37.9 | Sandy soil | Y | N |
| Gera River (Kenya) | (-0.44213889,  34.22666667) | 1198m | River | 30.4 | 8.3 | 79.1 | 39.4 | Sandy soil | Y | N |
| Luanda (Kenya) | (-0.46413889,  34.22916667) | 1172m | Stream | 29.2 | 7.5 | 39.2 | 16.4 | Silt soil | Y | N |
| Kulo Kudongo(Kenya) | (0.27072222,  34.19083333) | 1221m | Stream | 31.3 | 6.9 | 42.1 | 17.3 | Silt soil | Y | N |
| Wathi (Kenya) | (0.30513889,  34.23472222) | 1292m | River | 30.5 | 7.2 | 43.3 | 34.7 | Sandy soil | Y | N |
| Rakite (Kenya) | (0.32575,  34.19472222) | 1285m | Stream | 21.2 | 8.2 | 23.1 | 12.4 | Silt soil | Y | Y |
| Matodo (Kenya) | (0.30847222,  34.22972222) | 1261m | Stream | 31.3 | 7.3 | 42.1 | 13.6 | Silt soil | Y | N |
| Nyaitho (Kenya) | (-0.179, 35.07306) | 1293m | Stream | 29.1 | 7.3 | 37.9 | 12.6 | Silt soil | Y | N |
| Asawo (Kenya) | (-0.31817,  35.007) | 1232m | River | 24.5 | 6.8 | 28.6 | 23.7 | Silt soil | Y | Y |
| Awach (Kenya) | (-0.23405,  34.95717) | 1278m | River | 29.2 | 7.2 | 43.1 | 33.7 | Sandy soil | N | N |
| Awach B  (Kenya) | (-0.27203,  35.00374) | 1273m | River | 29.7 | 6.4 | 38.2 | 31.6 | Sandy soil | N | N |
| Asao A (Kenya) | (-0.29416,  34.94199) | 1255m | River | 30.1 | 7.2 | 41.2 | 35.2 | Sandy soil | N | N |
| Asao B (Kenya) | (-0.23403, 34.95717 | 1327m | River | 31.2 | 7.5 | 43.5 | 36.3 | Sandy soil | N | N |
| Agoro (Kenya) | (-0.27203,  35.00374) | 1225m | Stream | 30.6 | 7.5 | 42.1 | 12.2 | Silt soil | N | N |
| Nyando (Kenya) | (-0.17244,  34.92066) | 1155m | River | 31.2 | 7.3 | 53.8 | 35.6 | Sandy soil | N | N |
| Ahero (Kenya) | (-0.17656,  34.92103) | 1153m | Irrigation scheme | 30.6 | 7.3 | 29.1 | 2.1 | Silt soil | Y | N |
| Miriu (Kenya) | (-0.39753,  35.01759) | 1145m | River | 31.3 | 7.6 | 42.1 | 37.5 | Sandy soil | N | N |
| Omondo (Kenya) | (-0.30537,  34.93082) | 1142m | Stream | 30.6 | 7.3 | 39.1 | 15.2 | Silt soil | Y | N |
| Ochuoga (Kenya) | (-0.30075,  34.9295) | 1141m | Stream | 31.3 | 7.6 | 31.2 | 16.2 | Silt soil | Y | N |
| Kosele (Kenya) | (-0.44471,  34.68103) | 1330m | Stream | 29.8 | 7.4 | 33.6 | 11.3 | Silt soil | Y | N |
| Pundo (Kenya) | (-0.50641,  34.73087) | 1410m | Stream | 15.3 | 6.3 | 32.5 | 12.1 | Silt soil | Y | N |
| Kodumo (Kenya) | (-0.41068,  34.99455) | 1532m | Stream | 21.2 | 7.3 | 29.7 | 12.9 | Silt soil | Y | Y |
| Kodongo (Kenya) | (-0.444710, 34.681030) | 1548 | Stream | 16.2 | 6.9 | 26.7 | 15.4 | Silt soil | Y | Y |
| Katengu (Kenya) | (-0.39756,  35.00266) | 1547m | Stream | 15.2 | 7.3 | 32.1 | 18.3 | Silt soil | Y | N |
| K’otieno (Kenya) | (-0.51078,  34.71803) | 1544m | Stream | 13.2 | 7.2 | 31.5 | 13.5 | Silt soil | Y | N |
| Onsando (Kenya) | (-0.71103,  35.0479) | 1926m | Dam | 25.4 | 6.7 | 42.6 | 4.2 | Silt soil | Y | Y |
| Siwot (Kenya) | (-0.87596,  35.3683) | 2166m | Dam | 15.2 | 7.2 | 52.5 | 5.1 | Sandy soil | N | N |
| Cheptuyet  (Kenya) | (-0.91062,  35.34843) | 1914m | Stream | 25.3 | 8.3 | 27.3 | 14.1 | Silt soil | Y | Y |
| Kabere (Uganda) | (0.41284,  33.49619) | 1156m | Stream | 19.1 | 9.2 | 19.4 | 2.6 | Silt soil | Y | N |
| Kibimba (Uganda) | (0.52956,  33.85815) | 1080m | Irrigation scheme | 24.7 | 7.3 | 21.7 | 2.1 | Silt soil | Y | N |
| Macheche (Uganda) | (0.39348,  33.48281) | 1134m | Dam | 31.2 | 8.1 | 57.1 | 3.4 | Sandy soil | N | N |
| Kaitambiri (Uganda) | (1.12788,  33.70906) | 1083m | Stream | 15.2 | 8.4 | 32.5 | 18.3 | Silt soil | Y | N |
| Nakitende (Uganda) | (1.13265,  33.67626) | 1043m | Stream | 14.2 | 7.6 | 37.6 | 12.4 | Sandy soil | Y | N |
| Walukuba  (Uganda) | (0.44258,  33.22391) | 1139m | Stream | 25.6 | 7.3 | 26.3 | 14.2 | Silt soil | Y | Y |
| Ariet (Uganda) | (1.16151,  33.72042) | 1073m | Dam | 15.2 | 7.3 | 46.3 | 4.8 | Sandy soil | Y | N |
| Bisina (Uganda) | (1.59835,  33.95884) | 1043m | River | 14.2 | 7.4 | 52.4 | 32.6 | Sandy soil | Y | N |
| Opiyai (Uganda) | (1.70238,  33.62261) | 1119m | Spring | 25.2 | 6.2 | 27.2 | 12.6 | Silt soil | Y | Y |
| Amidakan (Uganda) | (1.93723,  33.34933) | 1043m | Dam | 16.2 | 6.5 | 41.2 | 4.9 | Sandy soil | N | N |
| Kachung (Uganda) | (1.89908,  32.97164) | 1045m | River | 15.2 | 7.4 | 38.6 | 21.5 | Sandy soil | N | N |
| Masindi (Uganda) | (1.69541,  32.09238) | 1034m | River | 19.2 | 6.7 | 21.7 | 23.6 | Silt soil | Y | N |
| Kole (Uganda) | (2.302313,  32.68811) | 1056m | River | 21.3 | 6.3 | 27.6 | 13.7 | Silt soil | Y | N |
| Masaka (Uganda) | (-0.33681,  31.71796) | 1249m | Stream | 15.3 | 6.2 | 35.7 | 16.7 | Silt soil | Y | N |
| Kagadi (Uganda) | (0.94023,  30.81403) | 1146m | Swamp | 13.2 | 8.2 | 39.8 | 4.2 | Silt soil | Y | N |
| Panyango (Uganda) | (2.52544,  31.46533) | 615m | River shore | 31.2 | 7.3 | 41.3 | 9.2 | Silt soil | Y | N |
| Muzizi (Uganda) | (0.87095,  30.72997) | 1178m | River | 21.1 | 9.2 | 29.5 | 12.5 | Silt soil | Y | N |
| Ntoroko (Uganda) | (1.05375,  30.53696) | 631m | River | 20.8 | 8.4 | 28.6 | 13.6 | Silt soil | Y | Y |
| Lubiri (Uganda) | (2.45857, 31.49975) | 632m | River | 22.1 | 7.3 | 28.3 | 16.4 | Silt soil | Y | Y |
| Rwizi (Uganda) | (-0.61686,  30.66828) | 1480m | River | 16.2 | 7.2 | 38.6 | 32.1 | Sandy soil | Y | N |
| Mpanga (Uganda) | (0.658889,  30.273528) | 1521m | River | 15,.2 | 7.3 | 53.9 | 12.4 | Silt soil | Y | N |
| Kasese (Uganda) | (0.17418,  30.08111) | 925m | River | 17.2 | 7.1 | 48.6 | 24.6 | Silt soil | Y | N |
| Wia-Amwon (Uganda) | (2.2595,  32.87927) | 1100m | Stream | 15.2 | 7.2 | 42.8 | 23.5 | Silt soil | Y | N |
| Mapea (Tanzania) | (-3.99006,  35.73814) | 962m | Irrigation scheme | 13.1 | 6.8 | 38.6 | 3.6 | Silt soil | Y | N |
| Njoro A (Tanzania) | (-3.32104,  37.27832) | 710m | Swamp | 16.3 | 7.3 | 31.2 | 2.6 | Silt soil | Y | N |
| Njoro B (Tanzania) | (-3.32598,  37.27805) | 713m | Stream | 14.3 | 6.1 | 31.7 | 6.1 | Silt soil | Y | N |
| Rao (Tanzania) | (-3.3381,  37.35776) | 715 | River | 15.2 | 8.5 | 42.1 | 28.5 | Silt soil | Y | N |
| Kikavu (Tanzania) | (-3.43637, 37.29482) | 702m | Irrigation scheme | 16.2 | 8.2 | 35.9 | 7.6 | Sandy soil | Y | N |
| Mabogini (Tanzania) | (-3.40061,  37.36256) | 684m | Irrigation scheme | 24.3 | 8.8 | 27.6 | 5.7 | Silt soil | Y | N |
| Chiwe (Tanzania) | (-6.21983,  36.74357) | 1249m | River | 15.2 | 7.3 | 29.6 | 34.3 | Sandy soil | Y | N |
| Ihanda (Tanzania) | (-6.24869,  36.71776) | 1329m | Stream | 15.2 | 7.2 | 32.7 | 16.2 | Silt soil | Y | N |
| Mhango (Tanzania) | (-6.24053,  37.5274) | 1015m | Stream | 14.2 | 8.5 | 35.2 | 15.3 | Silt soil | Y | N |
| Mvomero (Tanzania) | (-6.30143,  37.44623) | 1003m | River | 16.2 | 8.2 | 41.6 | 32.5 | Sandy soil | Y | N |
| Mkindu (Tanzania) | (-6.24803,  37.54699) | 1114m | River | 14.2 | 7.5 | 43.7 | 36.6 | Sandy soil | N | N |
| Mkindo (Tanzania) | (-6.26084,  37.55375) | 977m | Irrigation scheme | 15.2 | 7.2 | 42.4 | 4.1 | Silt soil | Y | N |
| Mlegeni (Tanzania) | (-6.89333,  37.07764) | 476m | Stream | 16.3 | 7.6 | 41.2 | 5.7 | Silt soil | Y | N |
| Dago (Tanzania) | (-6.8934,  37.07775) | 481m | Stream | 15.3 | 8.1 | 32.1 | 21.4 | Silt soil | Y | N |
| Zombo (Tanzania) | (-6.96513,  36.91566) | 533m | Irrigation scheme | 16.2 | 8.5 | 36.6 | 3.6 | Silt soil | Y | N |
| Tindiga (Tanzania) | (-6.89507,  37.0883) | 459m | Irrigation scheme | 16.2 | 8.2 | 37.6 | 6.7 | Silt soil | Y | N |
| Chabi (Tanzania) | (-7.31147,  36.52885) | 576m | Irrigation scheme | 16.3 | 8.7 | 36.6 | 4.2 | Silt soil | Y | N |
| Mwega (Tanzania) | (-7.31152,  36.52878) | 660m | Irrigation scheme | 13.2 | 8.1 | 37.4 | 4.9 | Silt soil | Y | N |
| Bwawani (Tanzania) | (-7.73513,  35.71792) | 1530m | Dam | 24.1 | 8.5 | 18.6 | 13.7 | Silt soil | Y | Y |
| Ilongo (Tanzania) | (-8.76628,  33.74477) | 1519m | Irrigation scheme | 17.2 | 7.1 | 32.7 | 12.3 | Silt soil | Y | N |
| Nsalagah (Tanzania) | (-8.88944,  33.57027) | 1813m | Stream | 16.3 | 7.6 | 37.5 | 15.3 | Silt soil | Y | N |
| Migombani (Tanzania) | (-9.31781,  32.76955) | 1654m | Stream | 32.2 | 7.3 | 34.6 | 13.1 | Silt soil | Y | N |
| Kasekera (Tanzania) | (-4.689278,  29.622083) | 1128m | Stream | 31.2 | 7.2 | 39.2 | 16.5 | Silt soil | Y | N |
| Singida (Tanzania) | (-4.788596, 34.745608) | 1388m | River | 31.2 | 7.5 | 43.2 | 12.5 | Sandy soil | N | N |
| Kibilizi (Tanzania) | (-4.860920, 29.624456) | 767m | Stream | 26.1 | 7.3 | 34.3 | 12.3 | Silt soil | N | Y |
| Musoma (Tanzania) | (-1.494590, 33.810970) | 1134m | River | 29.2 | 6.5 | 39.5 | 28.3 | Sandy soil | Y | N |
| Bweri (Tanzania) | (-1.537530, 33.854760) | 1250m | Stream | 14.7 | 6.9 | 43.2 | 12.4 | Silt soil | Y | N |
| Ziro ziro (Tanzania) | (-1.598370, 33.909480) | 1214m | Stream | 31.2 | 7.2 | 35.4 | 16.3 | Silt soil | Y | N |
| Musoma (Tanzania) | (-1.414650, 34.213500) | 1350m | Stream | 29.2 | 7.5 | 33.6 | 24.6 | Sandy soil | Y | N |
| Kongoro (Tanzania) | (-1.339650, 34.385780) | 1449m | Stream | 15.8 | 6.9 | 34.2 | 23.4 | Sandy soil | Y | N |
| Suguti (Tanzania) | (-1.680613, 33.703669) | 1197m | River | 16.5 | 7.1 | 45.2 | 29.2 | Sandy soil | Y | N |
| Nansimo (Tanzania) | (-2.157424, 33.443241) | 1141m | River | 16.3 | 7.9 | 32.6 | 13.5 | Sandy soil | Y | N |
| Lamadi (Tanzania) | (-2.246043, 33.836968) | 1140m | River | 15.3 | 7.2 | 38.6 | 16.4 | Sandy soil | Y | N |
| Mwamanyili (Tanzania) | (-2.372647, 33.563318) | 1155m | River | 14.3 | 7.1 | 45.3 | 16.9 | Silt soil | Y | N |
| Igogo (Tanzania) | (-2.539848, 32.901068) | 1134m | River | 29.3 | 6.8 | 35.2 | 15.8 | Silt soil | Y | N |
| Mnyuzi (Tanzania) | (-5.305361, 38.628585) | 230m | Stream | 31.2 | 6.5 | 32.5 | 15.6 | Silt soil | Y | N |
| Utwigu (Tanzania) | (-4.446061, 33.050964) | 1227m | Stream | 20.2 | 6.3 | 29.6 | 13.6 | Silt soil | Y | Y |
| Chigunga (Tanzania) | (-2.778439, 32.034190) | 1139m | River | 14.2 | 6.5 | 67.3 | 12.7 | Sandy soil | Y | N |
| Igombe (Tanzania) | (-4.888247, 32.754505) | 1149m | Dam | 28.3 | 7.2 | 58.5 | 2.6 | Sandy soil | N | N |
| Mwaseni (Tanzania) | (-7.792478, 38.094281) | 64m | River | 17.2 | 7.5 | 32.1 | 13.6 | Sandy soil | Y | N |
| Songea (Tanzania) | (-10.662715, 35.56577) | 932m | Dam | 15.5 | 6.9 | 57.3 | 21.8 | Sandy soil | Y | N |
| Masonya (Tanzania) | (-10.6992, 37.356541) | 527m | River | 15.4 | 8.1 | 32.5 | 13.4 | Sandy soil | Y | N |
| Shanwe (Tanzania) | (-6.32568, 31.052803) | 969m | Stream | 15.2 | 7.3 | 37.5 | 12.7 | Silt soil | Y | N |
| Sangari (Tanzania) | (-5.17815, 31.15624) | 1058m | River | 16.3 | 6.5 | 35.4 | 13.8 | Silt soil | N | N |
| Ntalikwa (Tanzania) | (-5.07492, 32.71561) | 1147m | Stream | 17.3 | 7.4 | 32.1 | 15.3 | Sandy soil | Y | N |
| Ntalikwa (Tanzania) | (-5.0742, 32.71541) | 1140m | Irrigation scheme | 16.7 | 7.1 | 39.1 | 12.6 | Silt soil | Y | N |
| Sumbawanga (Tanzania) | (-7.99843, 31.61841) | 846m | Stream | 15.6 | 6.7 | 43.2 | 15.4 | Silt soil | Y | N |
| Bujonde (Tanzania) | (-9.66072,  33.95171) | 482m | River | 30.4 | 7.2 | 38.1 | 23.6 | Sandy soil | Y | N |
| Matema (Tanzania) | (-9.49525,  34.02471) | 476m | River | 29.3 | 6.9 | 54.2 | 26.4 | Sandy soil | Y | N |
| Igalula (Tanzania) | (-5.63833,  32.62784) | 1123m | Stream | 28.1 | 7.4 | 37.2 | 14.5 | Silt soil | Y | N |
| Mwamgongo (Tanzania) | (-4.62742,  29.65162) | 676m | Stream | 25.9 | 6.8 | 28.1 | 13.7 | Silt soil | Y | Y |
| Bugamba (Tanzania) | (-4.56683,  29.65203) | 767m | Stream | 32.1 | 8.1 | 35.7 | 19.2 | Silt soil | Y | N |
| Kiziba (Tanzania) | (-4.51933, 29.66061) | 916m | Stream | 29.1 | 7.2 | 56.2 | 21.6 | Silt soil | Y | N |
| Babati (Tanzania) | (-4.227588, 35.744211) | 979m | River | 24.5 | 8.1 | 53.2 | 18.2 | Silt soil | Y | N |
| Mbaka (Tanzania) | (-9.549804, 33.957001) | 479m | River | 29.5 | 6.8 | 45.2 | 12.9 | Silt soil | Y | N |
| Mwaya (Tanzania) | (-9.558547, 33.948174) | 465m | Stream | 31.4 | 7.2 | 32.1 | 15.6 | Silt soil | Y | N |
| Galana (Tanzania) | (-2.201967, 38.058157) | 561m | River | 32.3 | 7.3 | 53.2 | 12.5 | Silt soil | Y | N |
| Mpanda (Tanzania) | (-6.355559, 31.053331) | 1751m | River | 29.3 | 7.1 | 65.3 | 13.4 | Silt soil | Y | N |
| Ibulwa (Tanzania) | (-5.215938, 32.669059) | 1167m | River | 31.4 | 6.8 | 56.2 | 12.9 | Silt soil | Y | N |
| Shama (Tanzania) | (-6.485610, 33.110264) | 1136m | River | 15.3 | 7.3 | 56.2 | 12.4 | Silt soil | Y | N |
| Lupa (Tanzania) | (-7.904587, 33.303941) | 1387m | River | 13.1 | 6.7 | 62.2 | 15.6 | Sandy soil | Y | N |
| Mindu (Tanzania) | (-6.868224, 37.613939) | 276m | Dam | 29.2 | 7.2 | 73.2 | 3.6 | Sandy soil | Y | N |
| Midiho (Tanzania) | (-5.538566, 32.590210) | 1173m | River | 17.3 | 7.5 | 65.2 | 11.6 | Sandy soil | Y | N |
| Kisisi (Tanzania) | (-5.244343, 32.707229) | 1196m | River | 15.3 | 7.9 | 73.2 | 12.5 | Sandy soil | Y | N |
| Duluti (Tanzania) | (**-**3.385123, 36.786488) | 1057m | Dam | 13.2 | 9.1 | 59.6 | 15.4 | Sandy soil | Y | N |
| Holili (Tanzania) | (-3.378823, 37.620629) | 883m | Stream | 17.3 | 8.3 | 39.2 | 19.2 | Silt soil | Y | N |
| Mkondoa (Tanzania) | (-6.825841, 37.161774) | 539m | River | 15.3 | 6.3 | 62.3 | 12.8 | Sandy soil | Y | N |
| Mkundi (Tanzania) | (-6.384851, 37.360174) | 412m | River | 17.6 | 7.1 | 54.2 | 16.9 | Silt soil | Y | N |
| Gairo (Tanzania) | (-6.127093, 36.867884) | 807m | Stream | 29.7 | 7.2 | 38.1 | 12.4 | Silt soil | Y | N |
| Lumuma (Tanzania) | (-7.333937, 36.521680) | 934m | River | 29.5 | 7.3 | 45.1 | 27.4 | Sandy soil | Y | N |
| Bomani (Tanzania) | (-1.345125, 34.372426) | 1419m | Stream | 31.2 | 7.1 | 38.2 | 23.7 | Silt soil | Y | N |
| Mori (Tanzania) | (-1.346410, 34.087468) | 1231m | River | 30.2 | 6.4 | 37.2 | 29.3 | Sandy soil | Y | N |
| Nkumbu (Tanzania) | (-3.695077, 33.460108) | 1183m | River | 31.2 | 7.9 | 35.1 | 21.9 | Silt soil | Y | N |
| Mori (Tanzania) | (-3.695077, 33.460108) | 1442m | River | 29.4 | 8.1 | 45.2 | 27.4 | Silt soil | Y | N |
| Nkaiti (Tanzania) | (-3.791196,  35.9164310) | 1001m | River | 31.2 | 9.2 | 53.1 | 12.3 | Sandy soil | Y | N |
| Pangani (Tanzania) | (-3.382294, 37.324104) | 834m | River | 30.8 | 7.4 | 45.2 | 18.3 | Sandy soil | Y | N |
| Nyankanga (Tanzania) | (-1.588600, 33.901086) | 1224m | Stream | 29.2 | 7.2 | 56.2 | 21.8 | Sandy soil | Y | N |

**Supplementary Figure 1:** Potential distribution of *B. pfeifferi* snails in Africa and East Africa, courtesy of MaxEnt. The dark red colour signifies locations with the highest probability of *B. pfeifferi* snails being found, followed by light red then orange, dark yellow, light yellow, dark green, light green, light blue, azure blue and finally dark blue with the lowest probability.


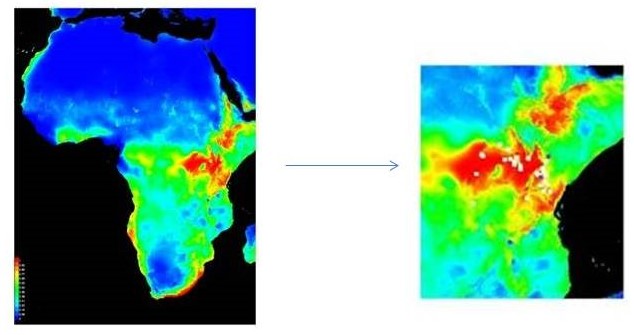


**Supplementary Figure 2**: Maximum likelihood phylogenetic tree based on sequences of the CO1 gene showing the evolutionary relationships among African *Biomphalaria* snails and the phylogenetic position of the snails collected during our fieldwork within this phylogeny. The tree is rooted on the South American taxon *B. glabrata.* GenBank reference sequences representing the African *Biomphalaria* species are highlited in bold and include the GenBank accession number. Sequences of snails generated during our study are labelled acording to collection locality and include country of origin in parentheses.


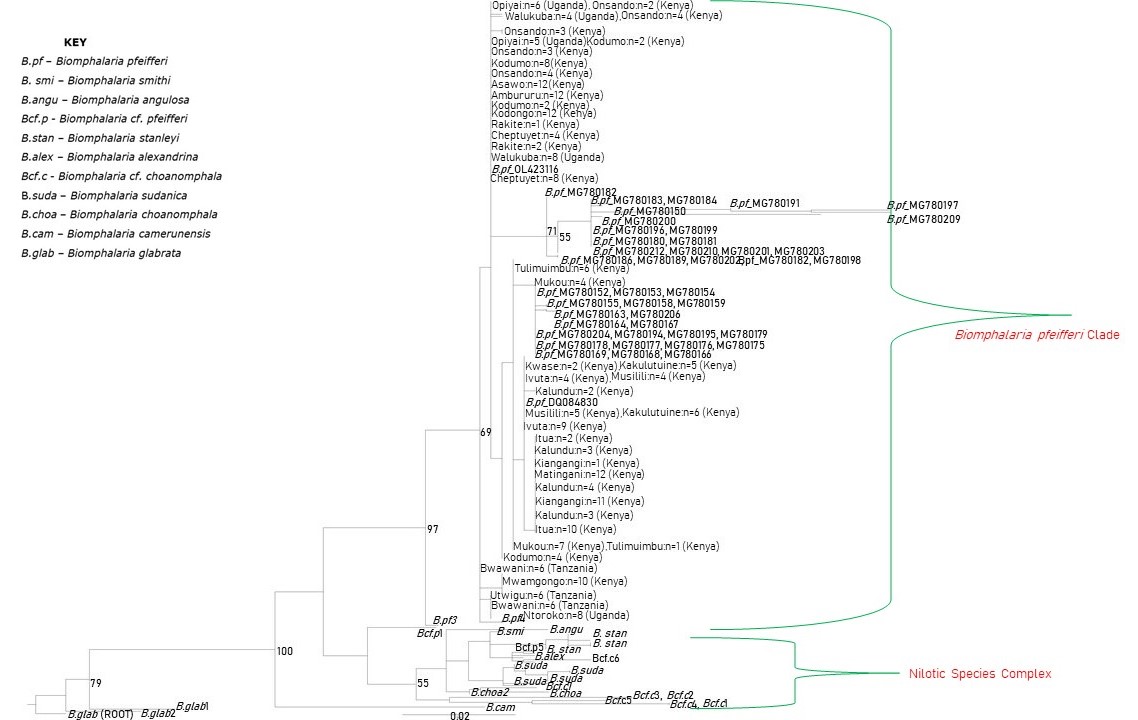


**Supplementary Figure 3:** A selection of *B. pfeifferi* shells from the East African collection sites.

| **Name of site** | **Coordinates** | **Altitude**  **(metres-m)** | **Type of habitat** | ***Biomphalaria* species found** | **Image of snail** |
| --- | --- | --- | --- | --- | --- |
| Matingani seepage (**Kenya**) | (-1.164, 38.005) | 1184m | Spring | *B. pfeifferi* | 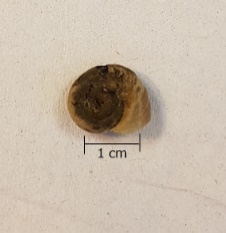 |
| Kiangangi irrigation canal (**Kenya**) | (-0.593833,  37.341972) | 1365m | Irrigation scheme | *B. pfeifferi* | 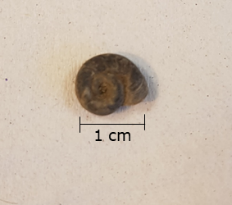 |
| Kalundu stream (**Kenya**) | (-1.35616667, 38.00611111) | 1216m | Stream | *B. pfeifferi* | 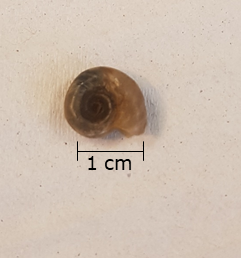 |
| Kwase stream (**Kenya**) | (-1.29883333,  37.35722222) | 1568m | Stream | *B. pfeifferi* | 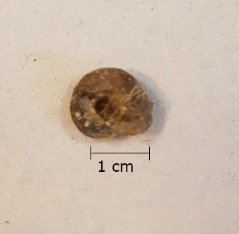 |
| Mutanga Ivuta stream (**Kenya**) | (-1.35758333,  37.35472222) | 1484m | Stream | *B. pfeifferi* | 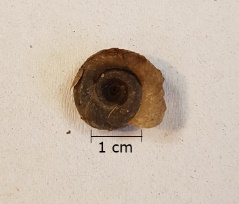 |
| Itua River (**Kenya**) | (-0.62888889,  37.54) | 653m | River | *B. pfeifferi* | 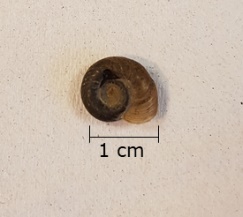 |
| Tulimiumbu stream (**Kenya**) | (-0.91133333,  37.65833333) | 1007m | Stream | *B. pfeifferi* | 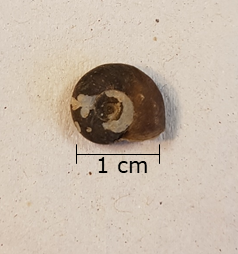 |
| Musilili stream (**Kenya**) | (-1.45023611,  37.2575) | 1330m | Stream | *B. pfeifferi* | 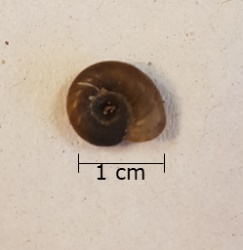 |
| Kakulutuine stream (**Kenya**) | (-1.20472222,  37.33055556) | 1188m | Stream | *B. pfeifferi* | 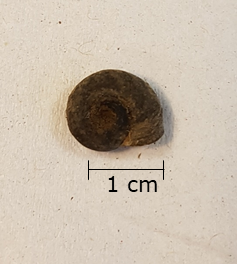 |
| Mukou stream (**Kenya**) | (-1.68488889,  37.34472222) | 1319m | Stream | *B. pfeifferi* | 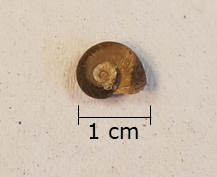 |
| Ambururu stream (**Kenya**) | (0.15163889,  34.27916667) | 1236m | Stream | *B. pfeifferi* | 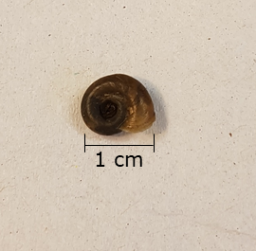 |
| Rakite stream (**Kenya**) | (0.32575,  34.19472222) | 1285m | Stream | *B. pfeifferi* | 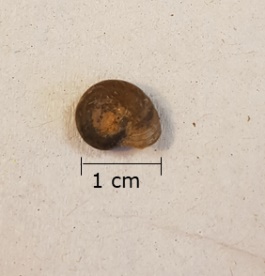 |
| Asawo River (**Kenya**) | (-0.31817,  35.007) | 1232m | River | *B. pfeifferi* | 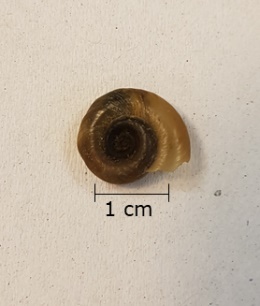 |
| Kodumo stream (**Kenya**) | (-0.41068,  34.99455) | 1532m | Stream | *B. pfeifferi* | 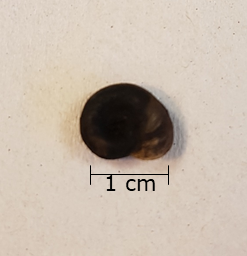 |
| Kodongo stream (**Kenya**) | (-0.444710, 34.681030) | 1548m | Stream | *B. pfeifferi* | 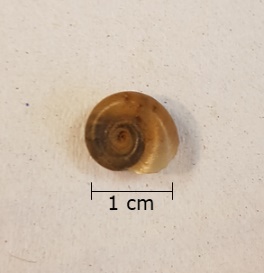 |
| Onsando Dam (**Kenya**) | (-0.71103,  35.0479) | 1926m | Dam | *B. pfeifferi* | 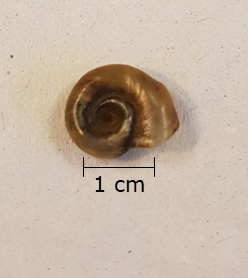 |
| Cheptuyet stream (**Kenya**) | (-0.91062,  35.34843) | 1914m | Stream | *B. pfeifferi* | 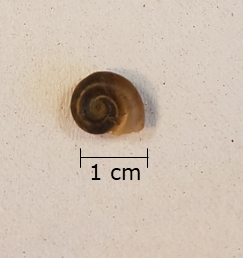 |
| Walukuba stream (**Uganda**) | (0.44258,  33.22391) | 1139m | Stream | *B. pfeifferi* | 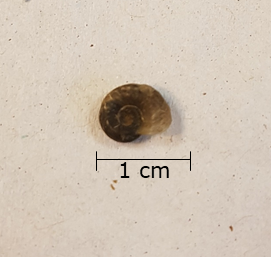 |
| Opiyai A Angorom (**Uganda**) | (1.70238,  33.62261) | 1119m | Spring | *B. pfeifferi* | 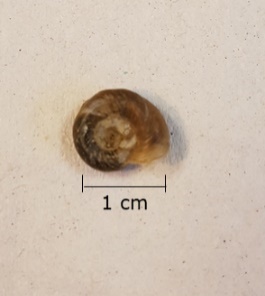 |
| Ntoroko (**Uganda**) | (1.05375,  30.53696) | 631m | River | *B. pfeifferi* | 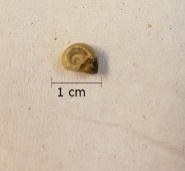 |
| Bwawani, Iringa (**Tanzania**) | (-7.73513,  35.71792) | 1530m | Dam | *B. pfeifferi* | 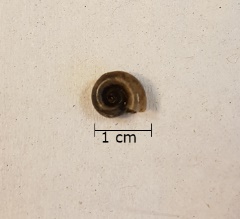 |
| Utwigu stream (**Tanzania**) | (-4.446061, 33.050964) | 1227m | Stream | *B. pfeifferi* | 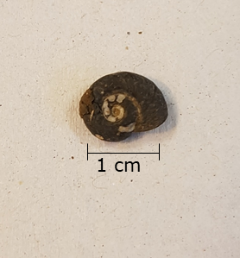 |
| Mwamgongo stream (**Tanzania**) | (-4.62742, 29.65162) | 676m | Stream | *B. pfeifferi* | 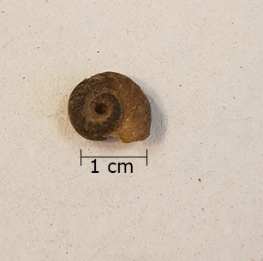 |

**Supplementary List 1:** List of GenBank data files for the CO1 sequences representing the *Biomphalaria* species used in constructing the phylogeny in Supplementary Figure 2.

*BIomphalaria pfeifferi*_MG780212.1

*Biomphalaria pfeifferi*_MG780211.1

*Biomphalaria pfeifferi*_MG780210.1

*Biomphalaria pfeifferi*_MG780209.1

*Biomphalaria pfeifferi*_MG780208.1

*Biomphalaria pfeifferi*_MG780207.1

*Biomphalaria pfeifferi*_MG780206.1

*Biomphalaria pfeifferi*_MG780205.1

*Biomphalaria pfeifferi*_MG780204.1

*Biomphalaria pfeifferi*_MG780203.1

*Biomphalaria pfeifferi*_MG780202.1

*Biomphalaria pfeifferi*_MG780201.1

*Biomphalaria pfeifferi*_MG780200.1

*Biomphalaria pfeifferi*_MG780199.1

*Biomphalaria pfeifferi*_MG780198.1

*Biomphalaria pfeifferi*_MG780197.1

*Biomphalaria pfeifferi*_MG780196.1

*Biomphalaria pfeifferi*_MG780195.1

*Biomphalaria pfeifferi*_MG780194.1

*Biomphalaria pfeifferi*_MG780193.1

*Biomphalaria pfeifferi*_MG780192.1

*Biomphalaria pfeifferi*_MG780191.1

*Biomphalaria pfeifferi*_MG780190.1

*Biomphalaria pfeifferi*_MG780189.1

*Biomphalaria pfeifferi*_MG780188.1

*Biomphalaria pfeifferi*_MG780187.1

*Biomphalaria pfeifferi*_MG780186.1

*Biomphalaria pfeifferi*_MG780185.1

*Biomphalaria pfeifferi*_MG780184.1

*Biomphalaria pfeifferi*_MG780183.1

*Biomphalaria pfeifferi*_MG780182.1

*Biomphalaria pfeifferi*_MG780181.1

*Biomphalaria pfeifferi*_MG780180.1

*Biomphalaria pfeifferi*_MG780179.1

*Biomphalaria pfeifferi*_MG780178.1

*Biomphalaria pfeifferi*_MG780177.1

*Biomphalaria pfeifferi*_MG780176.1

*Biomphalaria pfeifferi*_MG780175.1

*Biomphalaria pfeifferi*_MG780174.1

*Biomphalaria pfeifferi*_MG780173.1

*Biomphalaria pfeifferi*_MG780172.1

*Biomphalaria pfeifferi*_MG780171.1

*Biomphalaria pfeifferi*_MG780170.1

*Biomphalaria pfeifferi*_MG780169.1

*Biomphalaria pfeifferi*_MG780168.1

*Biomphalaria pfeifferi*_MG780167.1

*Biomphalaria pfeifferi_*MG780166.1

*Biomphalaria pfeifferi*_MG780165.1

*Biomphalaria pfeifferi*_MG780164.1

*Biomphalaria pfeifferi*_MG780163.1

*Biomphalaria pfeifferi*_MG780162.1

*Biomphalaria pfeifferi*_MG780161.1

*Biomphalaria pfeifferi*_MG780160.1

*Biomphalaria pfeifferi*_MG780159.1

*Biomphalaria pfeifferi*_MG780158.1

*Biomphalaria pfeifferi*_MG780157.1

*Biomphalaria pfeifferi*_MG780156.1

*Biomphalaria pfeifferi*_MG780155.1

*Biomphalaria pfeifferi*_MG780154.1

*Biomphalaria pfeifferi*_MG780153.1

*Biomphalaria pfeifferi*_MG780152.1

*Biomphalaria pfeifferi*_MG780151.1

*Biomphalaria pfeifferi*_MG780150.1

*Biomphalaria sp*._HM769258.1

*Biomphalaria sp._*HM769257.1

*Biomphalaria sp*._HM769256.1

*Biomphalaria sp*._HM769255.1

*Biomphalaria sp*._HM769254.1

*Biomphalaria sp*._HM769253.1

*Biomphalaria sp*._HM769252.1

*Biomphalaria sp*._HM769251.1

*Biomphalaria sp*._HM769250.1

*Biomphalaria sp.*_HM769249.1

*Biomphalaria sp*._HM769248.1

*Biomphalaria sp*._HM769247.1

*Biomphalaria sp*._HM769246.1

*Biomphalaria sp*._HM769245.1

*Biomphalaria sp*._HM769244.1

*Biomphalaria sp*._HM769243.1

*Biomphalaria sp*._HM769242.1

*Biomphalaria sp*._HM769241.1

*Biomphalaria sp*._HM769240.1

*Biomphalaria sp*._HM769239.1

*Biomphalaria sp*._HM769238.1

*Biomphalaria sp*._HM769237.1

*Biomphalaria sp*._HM769236.1

*Biomphalaria sp*._HM769235.1

*Biomphalaria sp*._HM769234.1

*Biomphalaria sp*._HM769233.1

*Biomphalaria sp*._HM769232.1

*Biomphalaria sp*._HM769231.1

*Biomphalaria sp*._HM769230.1

*Biomphalaria sp*._HM769229.1

*Biomphalaria sp*._HM769228.1

*Biomphalaria sp*._HM769227.1

*Biomphalaria sp*._HM769226.1

*Biomphalaria sp*._HM769225.1

*Biomphalaria sp*._HM769224.1

*Biomphalaria sp*._HM769223.1

*Biomphalaria sp*._HM769222.1

*Biomphalaria sp*._HM769221.1

*Biomphalaria sp*._HM769220.1

*Biomphalaria sp*._HM769219.1

*Biomphalaria sp*._HM769218.1

*Biomphalaria sp*._HM769217.1

*Biomphalaria sp*._HM769216.1

*Biomphalaria sp.*_HM769215.1

*Biomphalaria sp*._HM769214.1

*Biomphalaria sp*._HM769213.1

*Biomphalaria sp*._HM769212.1

*Biomphalaria sp*._HM769211.1

*Biomphalaria sp*._HM769210.1

*Biomphalaria sp*._HM769209.1

*Biomphalaria sp*._HM769208.1

*Biomphalaria sp*._HM769207.1

*Biomphalaria sp.*_HM769206.1

*Biomphalaria sp*._HM769205.1

*Biomphalaria sp*._HM769204.1

*Biomphalaria sp*._HM769202.1

*Biomphalaria sp*._HM769201.1

*Biomphalaria sp*._HM769200.1

*Biomphalaria sp*._HM769199.1

*Biomphalaria sp*._HM769198.1

*Biomphalaria sp*._HM769197.1

*Biomphalaria sp*._HM769196.1

*Biomphalaria sp*._HM769195.1

*Biomphalaria sp*._HM769194.1

*Biomphalaria sp*._HM769193.1

*Biomphalaria sp*._HM769192.1

*Biomphalaria sp*._HM769191.1

*Biomphalaria sp*._HM769190.1

*Biomphalaria sp*._HM769188.1

*Biomphalaria sp*._HM769187.1

*Biomphalaria sp*._HM769186.1

*Biomphalaria sp*._HM769185.1

*Biomphalaria sp*._HM769184.1

*Biomphalaria sp*._HM769183.1

*Biomphalaria sp*._HM769182.1

*Biomphalaria sp*._HM769181.1

*Biomphalaria sp*._HM769180.1

*Biomphalaria sp*._HM769179.1

*Biomphalaria sp*._HM769178.1

*Biomphalaria sp*._HM769176.1

*Biomphalaria sp*._HM769175.1

*Biomphalaria sp*._HM769174.1

*Biomphalaria sp*._HM769173.1

*Biomphalaria sp*._HM769172.1

*Biomphalaria sp*._HM769171.1

*Biomphalaria sp*._HM769170.1

*Biomphalaria sp*._HM769169.1

*Biomphalaria sp*._HM769168.1

*Biomphalaria sp*._HM769167.1

*Biomphalaria sp*._HM769166.1

*Biomphalaria sp*._HM769165.1

*Biomphalaria sp*._HM769164.1

*Biomphalaria sp*._HM769163.1

*Biomphalaria sp*._HM769162.1

*Biomphalaria sp*._HM769161.1

*Biomphalaria sp*._HM769160.1

*Biomphalaria sp*._HM769159.1

*Biomphalaria sp*._HM769158.1

*Biomphalaria sp*._HM769157.1

*Biomphalaria sp*._HM769156.1

*Biomphalaria sp*._HM769155.1

*Biomphalaria sp*._HM769154.1

*Biomphalaria sp*._HM769153.1

*Biomphalaria sp*._HM769152.1

*Biomphalaria sp*._HM769151.1

*Biomphalaria sp*._HM769150.1

*Biomphalaria sp*._HM769149.1

*Biomphalaria sp*._HM769148.1

*Biomphalaria sp*._HM769147.1

*Biomphalaria sp*._HM769146.1

*Biomphalaria sp*._HM769145.1

*Biomphalaria sp*._HM769144.1

*Biomphalaria sp*._HM769143.1

*Biomphalaria sp*._HM769142.1

*Biomphalaria sp*._HM769141.1

*Biomphalaria sp*._HM769140.1

*Biomphalaria sp*._HM769139.1

*Biomphalaria sp*._HM769138.1

*Biomphalaria sp*._HM769137.1

*Biomphalaria sp*._HM769136.1

*Biomphalaria sp*._HM769135.1

*Biomphalaria sp*._HM769134.1

*Biomphalaria sp*._HM769133.1

*Biomphalaria sp*._HM769132.1

*Biomphalaria choanomphala* _HM768906.1

*Biomphalaria choanomphala* _HM768905.1

*Biomphalaria sp*._HM768904.1

*Biomphalaria sp*._HM768903.1

*Biomphalaria sp*._HM768902.1

*Biomphalaria sp*._HM768902.1

*Biomphalaria choanomphala* _HM768949.1

*Biomphalaria choanomphala* _HM768948.1

*Biomphalaria choanomphala* _HM768947.1

*Biomphalaria choanomphala* _HM768946.1

*Biomphalaria choanomphala* _HM768945.1

*Biomphalaria choanomphala* _HM768944.1

*Biomphalaria choanomphala* _HM768943.1

*Biomphalaria choanomphala* _HM768942.1

*Biomphalaria choanomphala* _HM768941.1

*Biomphalaria choanomphala* _HM768940.1

*Biomphalaria choanomphala* _HM768939.1

*Biomphalaria choanomphala* _HM768938.1

*Biomphalaria choanomphala* _HM768937.1

*Biomphalaria choanomphala* _HM768936.1

*Biomphalaria choanomphala* _HM768935.1

*Biomphalaria choanomphala* _HM768934.1

*Biomphalaria choanomphala* _HM768933.1

*Biomphalaria choanomphala* _HM768932.1

*Biomphalaria choanomphala* _HM768931.1

*Biomphalaria choanomphala* _HM768930.1

*Biomphalaria choanomphala* _HM768929.1

*Biomphalaria choanomphala* _HM768928.1

*Biomphalaria choanomphala* _HM768927.1

*Biomphalaria choanomphala* _HM768926.1

*Biomphalaria choanomphala* _HM768925.1

*Biomphalaria choanomphala* _HM768924.1

*Biomphalaria choanomphala* _HM768923.1

*Biomphalaria choanomphala* _HM768922.1

*Biomphalaria choanomphala* _HM768921.1

*Biomphalaria choanomphala* _HM768919.1

*Biomphalaria choanomphala* _HM768918.1

*Biomphalaria choanomphala* _HM768917.1

*Biomphalaria choanomphala* _HM768916.1

*Biomphalaria choanomphala* _HM768915.1

*Biomphalaria choanomphala* _HM768914.1

*Biomphalaria choanomphala* _HM768913.1

*Biomphalaria choanomphala* _HM768912.1

*Biomphalaria choanomphala* _HM768911.1

*Biomphalaria choanomphala* _HM768910.1

*Biomphalaria choanomphala* _HM768909.1

*Biomphalaria choanomphala* _HM768908.1

*Biomphalaria choanomphala* _HM768907.1

*Biomphalaria sudanica* _AF199109.1

*Biomphalaria sudanica* _AF199108.1

*Biomphalaria sudanica* _AF199107.1

*Biomphalaria sudanica* _AF199106.1

*Biomphalaria pfeifferi*_AF199105.1

*Biomphalaria pfeifferi*_AF199104.1

*Biomphalaria pfeifferi*_AF199103.1

*Biomphalaria pfeifferi*_AF199102.1

*Biomphalaria pfeifferi*_AF199101.1

*Biomphalaria pfeifferi*_AF199100.1

*Biomphalaria pfeifferi*_AF199099.1

*Biomphalaria pfeifferi*_AF199098.1

*Biomphalaria pfeifferi*_AF199097.1

*Biomphalaria sudanica* _AF199088.1

*Biomphalaria sudanica* _DQ084844.1

*Biomphalaria sudanica* _DQ084843.1

*Biomphalaria cf. pfeifferi* (BpfMuz)_DQ084842.1

*Biomphalaria cf. pfeifferi* (BpfMas)_DQ084841.1

*Biomphalaria sudanica* _DQ084840.1

*Biomphalaria sudanica* _DQ084839.1

*Biomphalaria sudanica* _DQ084838.1

*Biomphalaria cf. pfeifferi* (BpfNga)_DQ084834.1

*Biomphalaria pfeifferi*_DQ084829.1

*Biomphalaria cf. pfeifferi* _DQ084841.1

*Biomphalaria cf. choanomphala* _EU141235.1

*Biomphalaria cf. choanomphala* _EU141234.1

*Biomphalaria cf. choanomphala* _EU141233.1

*Biomphalaria cf. choanomphala* _EU141226.1

*Biomphalaria cf. choanomphala* _EU141224.1

*Biomphalaria cf. choanomphala* _EU141223.1

*Biomphalaria cf. choanomphala* _EU141222.1

*Biomphalaria choanomphala* _DQ084828.1

*Biomphalaria sp*._KY745890.1

*Biomphalaria sp*._KY745889.1

*Biomphalaria sp*._KY745888.1

*Biomphalaria sp*._KY745887.1

*Biomphalaria sp*._KY745886.1

*Biomphalaria sp*._KY745885.1

*Biomphalaria sp*._KY745884.1

*Biomphalaria sp*._KY745883.1

*Biomphalaria sp*._KY745882.1

*Biomphalaria sp*._KY745881.1

*Biomphalaria sp*._KY745880.1

*Biomphalaria sp*._KY745878.1

*Biomphalaria sp*._KY745877.1

*Biomphalaria sp._*KY745876.1

*Biomphalaria sp._*KY745875.1

*Biomphalaria sp*._KY745874.1

*Biomphalaria sp._*KY745873.1

*Biomphalaria alexandrina* _DQ084825.1

*Biomphalaria alexandrina* _AF199110.1

*Biomphalaria alexandrina* _KF412766.1

*Biomphalaria smithi*_DQ084836.1

*Biomphalaria angulosa*_DQ084826.1

*Biomphalaria stanleyi*_EU141225.1

*Biomphalaria stanleyi*_EU141221.1

*Biomphalaria stanleyi*_EU141220.1

*Biomphalaria stanleyi*_EU141218.1

*Biomphalaria stanleyi*_EU141217.1

*Biomphalaria stanleyi*_EU141216.1

*Biomphalaria stanleyi*_EU141215.1
